# Green and Rapid Hydrothermal Crystallization and Synthesis of Fully Conjugated Aromatic Compounds

**DOI:** 10.1002/anie.201801277

**Published:** 2018-07-19

**Authors:** M. Josef Taublaender, Florian Glöcklhofer, Martina Marchetti‐Deschmann, Miriam M. Unterlass

**Affiliations:** ^1^ Institute of Applied Synthetic Chemistry TU Wien Getreidemarkt 9/163 1060 Wien Austria; ^2^ Institute of Materials Chemistry TU Wien Getreidemarkt 9/165 1060 Wien Austria; ^3^ Institute of Chemical Technologies and Analytics TU Wien Getreidemarkt 9/164 1060 Wien Austria

**Keywords:** carbonyl dyes, crystalline materials, fused heterocycles, green chemistry, hydrothermal synthesis

## Abstract

Highly fused, fully conjugated aromatic compounds are interesting candidates for organic electronics. With higher crystallinity their electronic properties improve. It is shown here that the crystallization of three archetypes of such molecules—pentacenetetrone, indigo, and perinone—can be achieved hydrothermally. Given their molecular structure, this is a truly startling finding. In addition, it is demonstrated that perinone can also be synthesized in solely high‐temperature water from the starting compounds naphthalene bisanhydride and *o*‐phenylene diamine without the need for co‐solvents or catalysts. The transformation can be drastically accelerated by the application of microwave irradiation. This is the first report on the hydrothermal generation of two fused heterocycles.

Organic colorants are molecules that show strong color when interacting with light. Aside from their oldest application, that is, coloring other substances, in recent years they have become of interest for optoelectronic applications in, for example, organic solar cells^1^ and as organic transistors.^2^ One important class of organic colorants are the so‐called carbonyl dyes, which are typically highly resistant to heat, solvents, and weathering.^3^ Perinone, that is, naphthalene tetracarboxylic acid bisbenzimidazole, is such a carbonyl dye. The name perinone is generally used for mixtures of *cis* and *trans* isomers (Figure 1).

**Figure 1:**
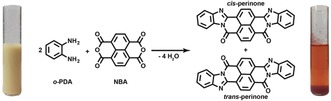
HT synthesis of perinone: *o*‐PDA and NBA undergo cyclocondensation to form a mixture of *cis*‐ and *trans*‐perinone. The initially white‐yellow suspension is transformed into a red solid and an orange, translucent supernatant.

These mixtures are of intense red color. The pure, separated isomers show cleaner, individual colors: *cis*‐perinone has a blueish‐red hue, while the industrially more important *trans*‐perinone has a brilliant orange‐reddish color.^4^ To the best of our knowledge, there is not a single direct synthetic approach that allows for obtaining exclusively either of the two isomers. This is because the isomers co‐crystallize: Indeed, the two isomers form a solid solution, which is structurally highly tolerant as reflected by the fact that the two isomers can be combined in a broad range of ratios by positional and rotational disorder.^5^ As in the last decades, research on perinone has been dominated by the development of isomer separation techniques rather than syntheses, there are only a handful of synthetic procedures towards perinone. These include:

1. refluxing the starting compounds in conc. acetic acid for several hours;^6^
2. condensation of the starting compounds in imidazole using Zn(OAc)_2_ as catalyst;^7^ this is analogous to the Langhals method for generating perylene bisimides;^8^
3. rapid condensation (15 min) in H_3_PO_4_ at 190 °C;^9^
4. precondensation to α‐amino‐imidazoles or α‐carboxylic acid imides in H_2_O at reflux, followed by solid‐state condensation to perinone.^10^

Clearly, all of the these syntheses except the latter two‐step route reported by Mamada et al. are far from being green approaches.^10^

With this contribution, we have set out to prepare perinone hydrothermally using nothing but high‐temperature water (HTW) and the starting compounds in a stoichiometric ratio. This work was prompted by our recent reports on the fully green hydrothermal (HT) synthesis of perylene and naphthalene bisimides,^11^ and polyimides.^12^, ^13^, ^14^ While others have used near‐critical water (250–350 °C) for the preparation of benzimidazoles,^15^ and supercritical water (≈400 °C) for various benzazoles,^16^ our HT method requires only HTW (typically 180–250 °C). Consequently, energy consumption is lower and the required safety measures are reduced. However, to date HT condensation has only been reported for the formation of single heterocycles and noncyclic amides.^12^, ^13^, ^14^, ^17^ In the latter cases, the action of HTW has been shown to generate superior crystallinity. Therefore, we were intrigued to expand the scope of HT synthesis and crystallization for the very first time towards fused heterocycles.

In an initial experiment, we investigated the general feasibility of hydrothermally generating perinone from the starting compounds *o*‐phenylene diamine (*o*‐PDA) and naphthalene bisanhydride (NBA), as shown in Figure 1. Therefore, *o*‐PDA and NBA (2:1 molar ratio at an equivalent concentration *c*_eq_=0.01 mol L^−1^) were suspended in deionized H_2_O and heated to 200 °C in a non‐stirred batch autoclave (see the Supporting Information (SI) for experimental details). After a reaction time *t*_R_ of 16 h, the autoclave contained two distinct phases: a red, flocculent solid as a sediment at the bottom of the glass liner, and an orange, translucent aqueous supernatant phase (see Figure 1). The sediment, whose red color was already indicative for the formation of perinone, was collected, dried and characterized without further purification. Attenuated total reflectance Fourier transform infrared (ATR‐FTIR) analysis (Figure 2; SI for details) of the crude product revealed the absence of modes characteristic for the starting compounds. Instead, we found several intense modes indicative for perinone.

**Figure 2:**
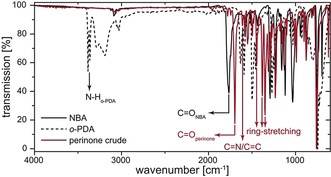
ATR‐FTIR spectra of crude perinone (red) and starting compounds (black).

The amount of obtained crude perinone corresponded to approximately 90 % of the theoretical yield. In order to further ascertain the formation of perinone, we performed solution ^1^H NMR analysis. ^1^H NMR measurements (Figure 3; SI) revealed, that the crude perinone is composed of a mixture of *cis* and *trans* isomers.^10^, ^18^ However, the two multiplets labeled as A and B in Figure 3 indicated the presence of a small amount of byproduct. Attempts to remove this byproduct via extraction were not successful. Therefore, we turned our attention to selective precipitation, a technique commonly used to purify or fractionate polydisperse polymers.^19^ By selective precipitation from trifluoroacetic acid (TFA) with H_2_O it was finally possible to purify the target compound and isolate the byproduct. The combined results of ATR‐FTIR, ^1^H NMR, and laser desorption/ionization high‐resolution mass spectrometry (LDI‐HRMS) measurements (see SI) served to identify the byproduct as naphthalene tetracarboxylic acid monoanhydride monobenzimidazole (NMM). Consequently, it was also possible to calculate the amount of NMM in crude perinone. According to ^1^H NMR analysis, NMM accounts for approximately 7 mol % of the unpurified product mixture (see SI for calculation). From these measurements the *cis*/*trans* ratio in the crude product as well as in reprecipitated perinone was determined to be approximately 2:3 (see SI for details).

**Figure 3:**
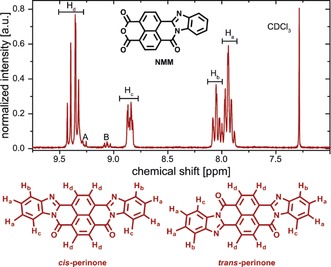
^1^H NMR spectrum of crude perinone and peak assignment for H_a_, H_b_, H_c_ and H_d_. Signals A and B can be attributed to the presence of the minor byproduct NMM.

A further set of experiments revealed that 1) a minimum of *t*_R_=12 h was necessary to yield maximum conversion of the starting compounds and 2) formation of NMM could not be avoided at any tested *t*_R_ (up to 48 h). Moreover, it became evident that NMM is formed as an intermediate during the reaction and its amount steadily decreases with *t*_R_ until a certain threshold is reached (after *t*_R_≥12 h).

The HT method for the condensation of *o*‐PDA and NBA described clearly qualifies as a green synthesis, since 1) no co‐solvents or catalysts are required and 2) H_2_O is not only the sole reaction medium but also the sole condensation byproduct. Nonetheless, we were interested in making the transformation even greener and more energy‐efficient. We therefore turned our attention towards microwave (MW)‐assisted HT condensation.^20^, ^21^ We conducted a series of experiments in a MW oven equipped with a stirrer, where it was possible to exactly control the reaction temperature *T*_R_, heating time *t*_H_ (time until *T*_R_ is reached), and *t*_R_ (reaction time at *T*_R_). When we performed these experiments at *T*_R_=200 °C and *t*_H_=10 min, we found that already after *t*_R_=2 min the starting compounds had completely reacted to form perinone. Evidently, not only efficient heating via MW irradiation, but also the application of stirring is highly beneficial for the reaction to proceed rapidly. Hence, through use of this MW setup it was possible to decrease the total reaction time at *T*_R_=200 °C from 12 h to 12 min. Lowering *T*_R_ to 180 °C drastically increases *t*_R_, whereas at the highest tested *T*_R_ of 250 °C it is not even necessary to maintain this *T*_R_ for a certain time. This tremendous reduction of *t*_R_ upon increasing *T*_R_ can be attributed, among other factors, to temperature‐dependent, significant changes in the physicochemical properties of H_2_O.^22^ With increasing *T*_R_ the static dielectric constant decreases steadily, which allows for better dissolution of organic compounds that are completely insoluble in H_2_O at RT. Furthermore, an elevation of *T*_R_ leads to an increasing ionic product of H_2_O until a maximum is reached at 250 °C. This phenomenon makes H_2_O itself a powerful acid/base catalyst in the HT regime, which is indeed beneficial for organic condensation reactions.

Interestingly, as long as full conversion of *o*‐PDA and NBA is achieved, the NMM content as well as the *cis*/*trans* ratio (≈2:3) remained constant independent of the reaction conditions. The typical amount of ≈7 mol % of NMM was always formed. Neither extending *t*_R_, increasing *t*_H_, varying the pH, nor lowering *c*_eq_ affected the ratio of isomers and the byproduct content. For the HT condensation of perylene and naphthalene bisanhydride with monoamines to yield bisimides it had been shown that the presence of a non‐nucleophilic base such as Hünig's base had a positive effect (reduction of *t*_R_, increase in yield) on the formation of some derivatives.^11^ Unfortunately, for the reaction of NBA with *o*‐PDA the addition of Hünig's base did not suppress or reduce the formation of NMM. One could furthermore speculate that NMM always forms due to a lack of *o*‐PDA, which could be consumed by the well‐known oxidative polymerization of aromatic amines.^12^ When we concentrated the liquid, orange supernatant phase, which was always found after the HT reaction, to dryness, a small amount of dark purple solid was obtained. FTIR‐ATR, ^1^H NMR, and ^13^C NMR analysis yielded spectra with a multitude of signals (SI), which likely correspond to various products of the oxidative autopolymerization of *o*‐PDA. In order to check whether the oxidative polymerization of *o*‐PDA is responsible for the formation of NMM, we employed a slight molar excess of *o*‐PDA. However, this did not result in the suppression of NMM formation. Clearly, in the absence of O_2_, oxidative polymerization of *o*‐PDA cannot occur. However, when perinone synthesis was performed after the reaction mixture had been thoroughly degassed, the usual amount of NMM was still obtained.

The fact that the formation of NMM is not suppressed under any of the tested conditions, including the addition of a condensation promoter and the use of excess *o*‐PDA, points to the incorporation of NMM into the crystal lattice of perinone as an alternative explanation. We suspect that the similar size and the planarity of NMM as well as its electronic similarity to *cis*‐ and *trans*‐perinone facilitates its incorporation into perinone's crystal lattice through positional and rotational disorder. Once NMM is incorporated, it can be considered as “entrapped” and consequently it is not available for further condensation. As for the curious fact that we always find ≈7 mol % of NMM in the crude perinone, we speculate that perinone crystals incorporate the amount of NMM that the crystal lattice can maximally tolerate.

Therefore, although the HT condensation of *o*‐PDA and NBA was found to be highly robust and proceeds rapidly at *T*_R_≥200 °C, selective precipitation from TFA was essential to remove traces of NMM. Interestingly, powder X‐ray diffraction (PXRD) measurements revealed that this purification technique significantly decreased crystallinity (Figure 4). In fact, reprecipitation with a non‐solvent rapidly quenches the perinone solution, thereby generating a strongly disordered liquid‐crystalline (LC) phase. When we attempted to index the reflections to a rectangular lattice, we found that all peaks fitted relatively well (see SI for detailed information) to a columnar rectangular LC phase.

**Figure 4:**
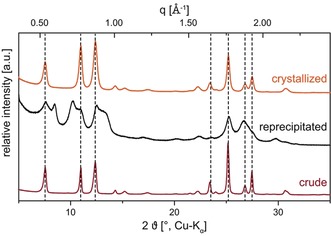
PXRD patterns of crude, reprecipitated, and crystallized perinone. Crude perinone (bottom) is highly crystalline, whereas the purified, reprecipitated perinone (middle) shows a LC type of ordering. Subsequent HT treatment leads to a highly crystalline product (top).

The unexpectedly high disorder in reprecipitated perinone provided an excellent opportunity for us to attempt the very challenging task of its HT crystallization. This task is ambitious, because perinone is much more hydrophobic than its precursors NBA and *o*‐PDA. Hence, greater difficulties regarding perinone's dissolution must be expected. At 250 °C polarity of H_2_O is comparable to that of ethanol at RT;^23^ thus H_2_O is certainly not apolar enough to expect it to be a good solvent for perinone.

To test the feasibility of HT crystallization, perinone was simply dispersed in H_2_O and heated to the HT regime. Fortunately, PXRD patterns showed that the HT treatment tremendously increased crystallinity (Figure 4). Furthermore, scanning electron microscopy (SEM) measurements clearly revealed that the HT treatment also improved the morphology (see SI): The reprecipitated roundish particles (0.1–0.5 μm) with a rough, random surface texture were transformed into agglomerates of fine needles (1–2 μm in length). When commercially available *trans*‐perinone was subjected to HT conditions, the morphological improvement was even more significant: beautiful needles (approximately 5–10 μm in length) with a rather narrow size distribution featuring smooth crystal facets were obtained (Figure 5).

**Figure 5:**
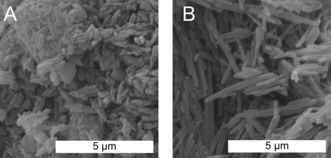
SEM images of *trans*‐perinone A) before and B) after HT treatment.

Hence, HT crystallization of perinone is indeed possible. These drastic morphological changes can only arise from HT dissolution of reprecipitated/commercially purchased perinone and its subsequent crystallization. Considering the polarity of H_2_O at 250 °C, it is very surprising that perinone dissolves. We suspect that the increased ionic product of HTW allows for the protonation of perinone, which is indeed the mechanism for its dissolution in some strong acids such as TFA. Additionally, the gain in lattice energy upon passing from the LC to the fully crystalline state could be a major driving force.

Thrilled by these findings, we decided to attempt the HT crystallization of other, fully conjugated aromatic compounds, also of interest for organic electronics and for which crystallinity matters. Specifically, we chose indigo (2,2′‐bis(2,3‐dihydro‐3‐oxoindolyliden)) and pentacenetetrone (pentacene‐5,7,12,14‐tetrone).^24^, ^25^ Like perinone, both of these compounds are insoluble in H_2_O at room temperature due to their ability to strongly π‐stack.^26^, ^27^ To test the feasibility of their HT crystallization, we applied the optimized reaction conditions found for crystallizing reprecipitated perinone. ATR‐FTIR and ^1^H NMR measurements revealed that both substances are stable under HT conditions (see SI). Furthermore, PXRD results indicated that dissolution and crystallization of these compounds is indeed possible under HT conditions, since reflections became sharper and more pronounced (see SI). SEM analysis (Figure 6; SI) additionally showed a striking improvement of the morphology for both compounds.

**Figure 6:**
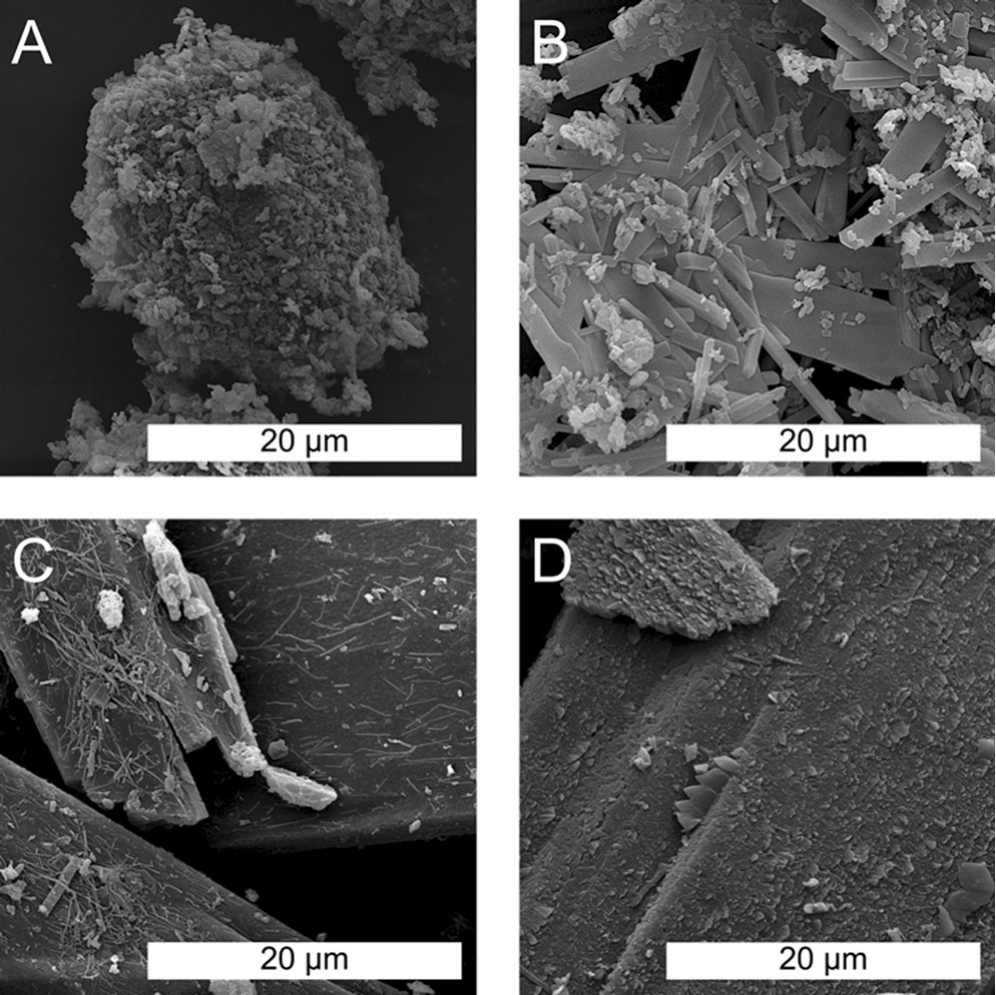
SEM images of indigo A) before and B) after HT treatment and pentacenetetrone C) before and D) after HT treatment.

In summary, we have shown that perinone can be synthesized hydrothermally solely from its starting compounds in stoichiometric ratio. The synthesis is highly robust and remains unaffected by various reaction parameters (*T*_R_, *t*_R_, *t*_H_, *c*_eq_, pH, O_2_) and does not require condensation promotors or catalysts. The obtained product only contains minor amounts of NMM byproduct. Moreover, we demonstrate that it is possible to crystallize intentionally amorphisized perinone as well as recrystallize commercially obtained perinone. Furthermore, we could show that HT crystallization can also be successfully applied to other fully conjugated aromatic compounds. These crystallization experiments set the basis for our strong conviction that HTW is a promising, cheap, and environmentally benign medium for crystallizing temperature‐stable low‐molecular‐weight compounds.

## Conflict of interest

The authors declare no conflict of interest.

## Supporting information

As a service to our authors and readers, this journal provides supporting information supplied by the authors. Such materials are peer reviewed and may be re‐organized for online delivery, but are not copy‐edited or typeset. Technical support issues arising from supporting information (other than missing files) should be addressed to the authors.

Supplementary
