# Supplementary material for: Green and Rapid Hydrothermal Crystallization and Synthesis of Fully Conjugated Aromatic Compounds
Source: Angew Chem Int Ed Engl. 2018 Jul 19;57(38):12270–4. doi: 10.1002/anie.201801277 (PMC6485404; doi:10.1002/anie.201801277)
Supplement: Supplementary file 1 — Supplementary [file ANIE-57-12270-s001.pdf]

Angewandte Chemie
Eine Zeitschrift der Gesellschaft Deutscher Chemiker

Supporting Information

Green and Rapid Hydrothermal Crystallization and Synthesis of Fully Conjugated Aromatic Compounds

*M. Josef Taublaender, Florian Glöcklhofer, Martina Marchetti-Deschmann, and Miriam M. Unterlass**

anie_201801277_sm_miscellaneous_information.pdf

Table of Contents

1 Methods 2

2 Chemicals 2

3 Experimental Details 3
- 3.1 Preliminary Experiments in Non-Stirred Batch Autoclaves 3
- 3.2 Experiments in Stirred Microwave-Assisted Batch Autoclaves 3
- 3.3 Purification of Perinone 5
  - 3.3.1 Attempts to Remove Byproduct *via* Extraction 5
  - 3.3.2 Removal of Byproduct *via* Selective Precipitation 5
- 3.4 LDI HRMS Preparation 6
- 3.5 Crystallization of Perinone, Indigo and Pentacenetetrone 6

4 Analysis and Characterization 8
- 4.1 ATR-FTIR Measurements 8
  - 4.1.1 Crude Perinone and Starting Compounds 8
  - 4.1.2 Reprecipitated Perinone and Byproduct 9
  - 4.1.3 Incomplete Perinone 11
  - 4.1.4 Oxidative Polymerization Products in Aqueous Supernatant 12
  - 4.1.5 Crystallized Perinone 12
- 4.2 NMR Measurements 14
  - 4.2.1 Crude Perinone 14
  - 4.2.2 Byproduct 16
  - 4.2.3 Reprecipitated Perinone 16
  - 4.2.4 Incomplete Perinone 18
  - 4.2.5 Oxidative Polymerization Products in Aqueous Supernatant 19
  - 4.2.6 Crystallized Perinone 20
  - 4.2.7 *trans*-Perinone 21
  - 4.2.8 Determining the Byproduct Content in Crude Perinone 23
- 4.3 LDI HRMS Measurements 24
- 4.4 PXRD Measurements 25
  - 4.4.1 Crude Perinone 25
  - 4.4.2 Reprecipitated Perinone 26
  - 4.4.3 Crystallized Perinone 29
  - 4.4.4 *trans*-Perinone 30
- 4.5 SEM Measurements 31
  - 4.5.1 Crude Perinone 31
  - 4.5.2 Reprecipitated Perinone 32
  - 4.5.3 Crystallized Perinone 32
  - 4.5.3 *trans*-Perinone Before Crystallization 33
  - 4.5.6 *trans*-Perinone After Crystallization 33

5 Crystallization of Indigo and Pentacenetetrone 34

6 References 40

1 Methods

Attenuated total reflectance Fourier transform infrared (ATR-FTIR) spectra were recorded on a Bruker Tensor 27 working in ATR MicroFocusing MVP-QL with a diamond crystal. Resolution was set to 4 cm^{-1} and spectra were recorded from 4000 cm^{-1} to 600 cm^{-1}.

^{1}H and ^{13}C solution nuclear magnetic resonance (NMR) spectra were recorded on a Bruker Avance DRX-400 spectrometer (400 MHz).

Laser desorption/ionization high resolution mass spectrometry (LDI HRMS) was performed on a Synapt G2 (Waters, Manchester, UK) with a MALDI ion source. The laser power was set to 275 a.u. and mass spectra were acquired at 20 – 1000 amu. High mass accuracy was achieved by using an appropriate lock-mass (m/z 379.0930) after external calibration (m/z 172.0399 –1673.9319).

Powder X-ray diffraction (PXRD) data was collected with a PANalytical X'Pert Pro multipurpose diffractometer (MPD) in Bragg Brentano geometry operating with a Cu anode at 40 kV, 40 mA. An X-Celerator multichannel detector was used. Samples were ground and mounted as loose powders on silicon single crystal sample holders. The diffraction patterns were recorded between 5 ° and 60 ° (2θ) with 69.215 s/step and a step size of 0.0050134 °. Sample holders were rotated during the measurement with 4 s/turn.

Scanning electron microscopy (SEM) was carried out with a Quanta 200F FEI microscope. Typically, the samples were measured at 10 kV with a working distance of 7 - 9 mm and spot size 2.0. Prior to imaging, samples were loaded on carbon-coated stubs and coated by sputtering with a 17 nm thick layer of Au/Pd 60/40 alloy with a Quarum Q105T S sample preparation system.

2 Chemicals

o-Phenylene diamine (*o*-PDA, >98%) was purchased as its dihydrochloride from TCI. Alkaline extraction with ethyl acetate yielded the free diamine *o*-PDA. Naphthalene bisanhydride (NBA, >97%) was purchased from TCI and had to be purified prior to use. The commercially available NBA contained traces of acetic acid (clearly visible in ^{1}H-NMR spectrum) and probably other, inorganic impurities. In order to remove them 5 g of NBA were dissolved in 250 mL of 1 M NaOH at room temperature (rt). While cooling in an ice bath naphthalene tetracarboxylic acid (NTCA) was precipitated *via* dropwise addition of diluted HCl. The residual solution had an intense yellow color indicating that certain impurities were still dissolved. The obtained white, solid product (various hydrates of NTCA) was isolated *via* centrifugation and dried under high vacuum conditions at 160 °C using an oil bath. As evinced by ^{1}H-NMR analysis, the finally obtained beige NBA did not contain acetic acid anymore. *trans*-Perinone (Pigment Orange 43, >94%), indigo (>97%) and pentacenetetrone (>94%) were purchased from TCI and used as received for crystallization experiments.

3 Experimental Details

3.1 Preliminary Experiments in Non-Stirred Batch Autoclaves

In order to perform the hydrothermal (HT) condensation of NBA and *o*-PDA to yield the target compound perinone both starting compounds (40.2 mg NBA and 32.5 mg *o*-PDA, molar ratio of 1:2, c_{eq} = 0.01 mol/L) were first placed in a glass liner (V_{liner} = 27 mL). 15 mL of distilled H_2O were added and the starting compounds were thoroughly dispersed by intense stirring at rt. After removing the stir bar the liner was placed into a PTFE-lined, non-stirred batch autoclave ($V_{autoclave}$ = 45 mL). The autoclave was put into an oven, which was preheated to the reaction temperature T_R of 200 °C, and kept there for the desired reaction time t_R. The experimental conditions of all performed autoclave experiments are listed in Table S 1. To stop the reaction the autoclave was quickly cooled to rt by quenching with cold tap H_2O. The obtained phases (a red solid on bottom and an orange, translucent liquid above) were separated *via* filtration and the obtained solid phase was washed with distilled H_2O and dried in a vacuum desiccator over silica gel blue.

Table S 1: Conditions for preliminary experiments carried out in non-stirred batch autoclaves at c_{eq} = 0.01 mol/L.

number	T_R [°C]	t_R [h]	comment
1	200	16	complete
2	200	2	incomplete
3	200	4	incomplete
4	200	8	incomplete
5	200	12	complete
6	200	48	complete

3.2 Experiments in Stirred Microwave-Assisted Batch Autoclaves

Both starting compounds (107.3 mg NBA and 86.6 mg *o*-PDA, molar ratio of 1:2, *ceq* = 0.01 mol/L) were placed in a glass liner (V_{liner} = 60 mL). 40 mL of distilled H_2O were added. The starting compounds were dispersed at rt under intense stirring before the liner was placed in a PTFE-lined, stirred batch microwave (MW) vessel (V_{vessel} = 120 mL). The dispersion was stirred while heating by MW irradiation to the intended T_R at a defined heating rate. Heating times t_H (time until T_R is reached), reaction times t_R (time for which T_R is held constantly) as well as certain deviations from the standard procedure are given for all experiments in Table S 2. At the end of the reaction the autoclave was allowed to slowly cool back to rt. Due to the experimental set-up quenching was not possible. Cooling down to rt took approximately 60 min from 200 °C or 80 min from 250 °C. The obtained solid phases were isolated, washed with distilled H_2O and dried in a vacuum desiccator over silica gel blue.

The above-mentioned deviations from the standard procedure include:

- Variation of c_{eq}
- Variation of pH by adding HCl (aq.) or NH_3 (aq.)
- Addition of HÜNIG's base (c_{eq} = 3 mmol/L) to the dispersion of starting compounds
- Employing an excess (3 eq. instead of 2 eq.) of *o*-PDA
- Degassing of the dispersion of starting compounds prior to the MW experiment by bubbling thoroughly with Ar for 15 min or by applying three consecutive freeze-thaw cycles

Table S 2: Conditions for experiments carried out in stirred MW-assisted batch autoclaves.

number	T_R [°C]	t_H [min]	t_R [min]	c_{eq} [mol/L]	other	comment
1	200	10	0	0.01	-	incomplete
2	200	10	2	0.01	-	complete
3	150	10	240	0.01	-	incomplete
4	180	10	60	0.01	-	incomplete
5	180	10	120	0.01	-	complete
6	250	10	0	0.01	-	complete
7	250	10	120	0.01	-	complete
8	250	60	0	0.01	-	complete
9	250	10	0	0.01	pH = 4	incomplete
10	250	10	15	0.01	pH = 4	complete
11	250	10	0	0.01	pH = 10	complete
12	250	10	0	0.005	-	complete
13	250	10	0	0.03	-	incomplete
14	250	10	15	0.03	-	complete
15	250	10	15	0.05	-	complete
16	250	10	0	0.01	HÜNIG's base	complete
17	250	10	0	0.01	3 eq. *o*-PDA	complete
18	250	10	0	0.01	Ar-bubbling	complete
19	250	10	0	0.01	freeze-thaw cycles	complete

An exemplary *T*-*t* profile of a MW-assisted reaction (where T_R = 250 °C, t_H = 10 min and t_R = 2 min) is shown in Figure S 1. Since the set-up we used did not allow for controlling the cooling rate, we could not study the parameter cooling time t_C. Hence, t_C and the cooling curve had to be estimated.

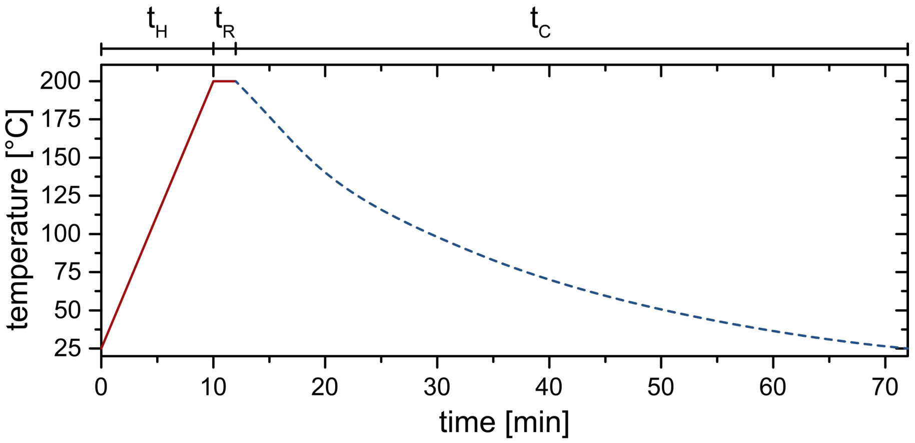

Figure S 1: Exemplary *T*−*t* profile of a MW-assisted perinone synthesis: characteristic times t_H, t_R and t_C are indicated. t_H and t_R could be controlled exactly, whereas t_C and the cooling curve could only be estimated.

3.3 Purification of Perinone

3.3.1 Attempts to Remove Byproduct *via* Extraction

Approximately 10 mg of finely ground crude perinone were placed in a flask and 10 mL of solvent (petroleum ether, ethyl acetate, acetone, dichloromethane, acetonitrile, toluene, MeOH, EtOH, DMF, DMSO, 1 M aqueous HCl or 1 M aqueous KOH, respectively) were added. In one set of experiments, the slurry was intensively stirred at rt overnight. In another set of experiments the mixture was heated while stirring for 1 h. For none of the tested solvents any dissolution was observed. Perinone was subsequently isolated *via* centrifugation. In order to remove high boiling solvents (DMF, DMSO) or traces of HCl and KOH prior to analysis, the corresponding solids were washed intensively with distilled H_2O multiple times. The different samples were dried in a vacuum desiccator over silica gel blue and analyzed by ^{1}H-NMR and ATR-FTIR spectroscopy. Regarding purity, no changes were observed.

3.3.2 Removal of Byproduct *via* Selective Precipitation

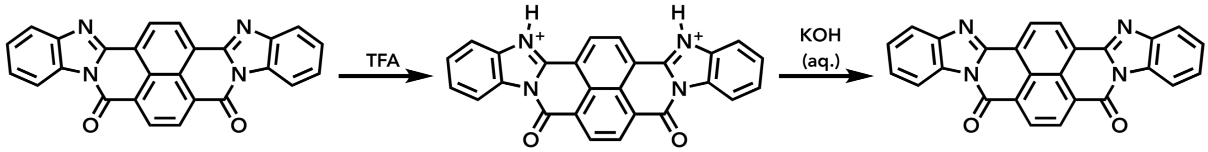

500 mg of crude perinone were dissolved in 50 mL trifluoroacetic acid (TFA). Afterwards, the obtained dark red solution was cooled in an ice bath. Slow and stepwise addition of 50 mL distilled H_2O led to precipitation of pure perinone. H_2O was added dropwise in portions of 1 mL to the non-stirred solution. After addition of each portion of H_2O, the solution was shaken carefully before the next portion of H_2O was added. The finally obtained phases (a dark red solid and a dark red liquid) were separated *via* centrifugation. The solid product was intensively stirred in 0.5 M aqueous KOH at rt for 10 min in order to ensure complete deprotonation. After a subsequent washing step with distilled H_2O the obtained powder was dried in a

vacuum desiccator over silica gel blue. ^{1}H-NMR and ATR-FTIR analysis confirmed complete removal of the byproduct (later identified as NMM; see manuscript and section 4). The liquid phase was evaporated to dryness and the obtained solid was intensively washed with aqueous KOH and subsequently distilled H_2O to yield the isolated byproduct fraction after desiccation.

This procedure is strongly exothermic and has to be performed extremely carefully. The TFA solution should be placed in the ice bath for at least 5 min prior to addition of H_2O.

3.4 LDI HRMS Preparation

0.3 mg of the isolated byproduct fraction were suspended in $CHCl_3$ and subsequently centrifuged at 12 000 xg. After evaporation of $CHCl_3$ 500 µL TFA were added and the byproduct was entirely dissolved *via* sonication. 1 µL sample was applied on a stainless-steel sample holder, dried under ambient conditions and directly measured by LDI HRMS.

3.5 Crystallization of Perinone, Indigo and Pentacenetetrone

165 mg of reprecipitated perinone (after purification *via* selective precipitation, c_{eq} = 0.01mol/L) were placed in a glass liner (V_{liner} = 60 mL). 40 mL of distilled H_2O were added and the dark red powder was dispersed at rt under intense stirring before the liner was placed in a PTFE-lined, stirred batch MW vessel (V_{vessel} = 120 mL). The dispersion was stirred while heating by MW irradiation to a T_R of 250 °C at a t_H of 10 min and held constantly at this T_R for t_R = 15 min and 60 min, respectively. After slowly cooling back to rt the obtained red solid was isolated *via* filtration from the completely clear supernatant, washed with distilled H_2O and dried in a vacuum desiccator over silica gel blue.

Crystallization experiments with commercially available *trans*-perinone, indigo and pentacenetetrone were carried out accordingly. For reasons of better comparability, the optimized reaction conditions found for reprecipitated perinone were also applied for these crystallization experiments.

The exact reaction conditions for all crystallization experiments are summarized in Table S 3.

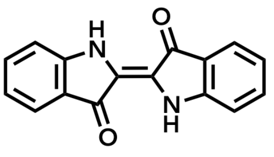

indigo

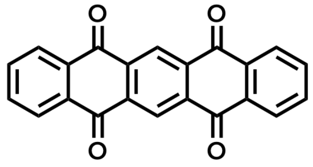

pentacenetetrone

Table S 3: Conditions for crystallization experiments with reprecipitated perinone, *trans*-perinone, indigo and pentacenetetrone.

compound	T_R [°C]	t_H [min]	t_R [min]	c_{eq} [mol/L]	m [mg]
reprecipitated perinone	250	10	15<br>60	0.01	165
trans-perinone	250	10	15	0.01	165
indigo	250	10	15	0.01	105
pentacene-tetrone	250	10	15	0.01	135

4 Analysis and Characterization

4.1 ATR-FTIR Measurements

4.1.1 Crude Perinone and Starting Compounds

In Figure S 2 the ATR-FTIR spectra of crude perinone (synthesized in a non-stirred batch autoclave at T_R = 200 °C for t_R = 16 h) and the starting compounds *o*-PDA and NBA are shown. For better clarity Figure S 3 depicts the ATR-FTIR spectrum solely of crude perinone. We conclude full conversion of the starting compounds, since their typical modes (*o*-PDA: ν_s(N-H) ≈ 3390 cm^{-1}, ν_{as}(N-H) ≈ 3360 cm^{-1}; NBA: ν(C=O)$_{NBA}$ ≈ 1765 cm^{-1}) are not visible anymore. Instead a whole new set of intense modes has evolved, indicating the presence of perinone: most prominently ν(C=O)$_{perinone}$ ≈ 1700 cm^{-1} and ν(C=N/C=C) ≈ 1610 cm^{-1},[i,ii] and also several highly characteristic benzimidazole ring-stretching modes (1450 cm^{-1}, 1380 cm^{-1} and 1355 cm^{-1}).[iii]

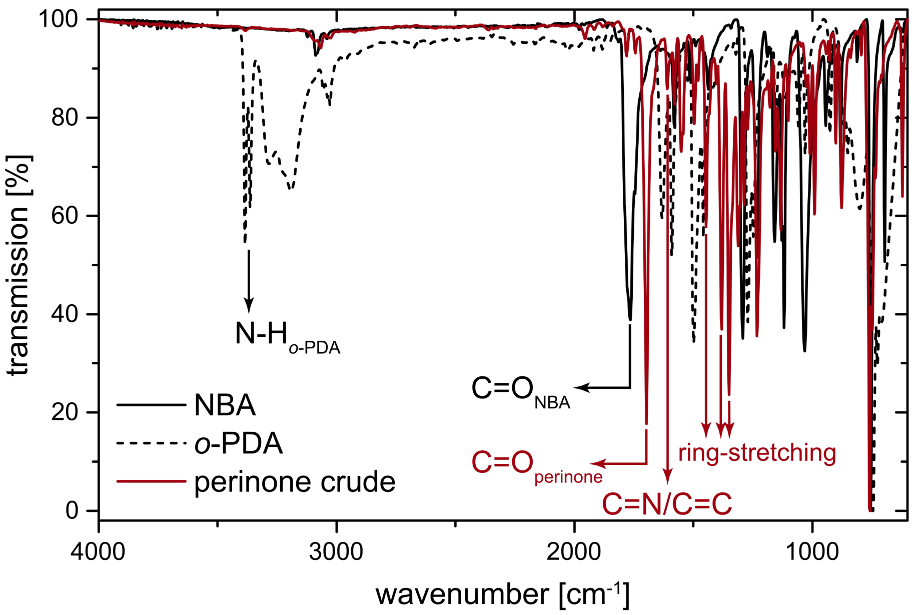

Figure S 2: Superimposed ATR-FTIR spectra of crude perinone (synthesized in a non-stirred batch autoclave at T_R = 200 °C for t_R = 16 h) and the starting compounds *o*-PDA and NBA: characteristic modes of the different compounds are labeled. The absence of characteristic modes of NBA and *o*-PDA (highlighted by black arrows) in the spectrum of crude perinone is an indication for full conversion. Moreover, the crude product's spectrum shows modes (highlighted by red arrows) that are highly indicative for perinone formation, namely ν(C=O)$_{perinone}$ ≈ 1700 cm^{-1}, ν(C=N/C=C) ≈ 1610 cm^{-1}, and benzimidazole ring-stretching modes (1450 cm^{-1}, 1380 cm^{-1} and 1355 cm^{-1}).

The FTIR-ATR spectra of all other crude samples, where maximum conversion of starting compounds was achieved (see Table S 1 and Table S 2), are completely identical to Figure S 3 independent of the experimental set-up used for their preparation (non-stirred batch autoclave or stirred MW-assisted batch autoclave).

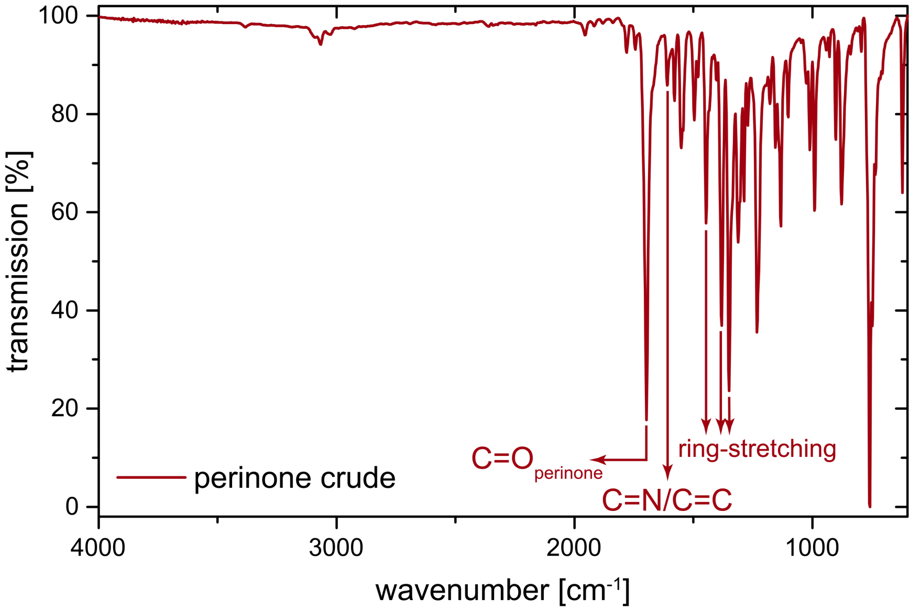

Figure S 3: ATR-FTIR spectrum of crude perinone (synthesized in a non-stirred batch autoclave at T_R = 200 °C for t_R = 16 h): characteristic perinone modes are highlighted: ν(C=O)$_{perinone}$ ≈ 1700 cm^{-1}, ν(C=N/C=C) ≈ 1610 cm^{-1} and the characteristic benzimidazole ring-stretching modes (1450 cm^{-1}, 1380 cm^{-1} and 1355 cm^{-1}).

4.1.2 Reprecipitated Perinone and Byproduct

The ATR-FTIR spectrum of pure perinone (obtained *via* selective precipitation) is depicted in Figure S 4 and only differs slightly from the one of the crude product (Figure S 2): the weak modes at 1780 cm^{-1} and 1760 cm^{-1} are missing.
In addition to the ATR-FTIR spectrum of pure perinone, Figure S 4 also shows the spectrum of the byproduct (NMM) separated by selective precipitation. The following characteristic modes (indicated by black arrows in Figure S 4) are present in both spectra - for pure perinone as well as for the obtained byproduct: ν(C=O)$_{perinone}$ ≈ 1700 cm^{-1}, ν(C=N/C=C) ≈ 1610 cm^{-1} and characteristic benzimidazole ring-stretching modes (1450 cm^{-1}, 1380 cm^{-1} and 1355 cm^{-1}). This already indicates for the byproduct a structure closely related to perinone, but also some additional modes can be found. The most important, indicative differences between the two spectra (highlighted by blue boxes and letters *a* and *b* in Figure S 4 B) are: *(i)* The byproduct contains modes at 1780 cm^{-1} and 1760 cm^{-1}, which lie in the region of C=O stretching modes for anhydrides and are also found in NBA (labeled as *a*); *(ii)* and a mode at 1035 cm^{-1} (labeled as *b*), which is not only present, but also highly pronounced in NBA's ATR-FTIR spectrum.
Furthermore, the modes at 2855 cm^{-1} and 2925 cm^{-1}, the broad mode in the range of 2950 cm^{-1} to 3600 cm^{-1} as well as the mode at 1660 cm^{-1} (shoulder of the intense perinone C=O mode at 1700 cm^{-1}) are a lot more pronounced for the byproduct compared to pure and crude perinone, respectively.
The combined results of various analytical techniques (ATR-FTIR, ^{1}H-NMR and LDI HRMS) finally allowed to identify the byproduct as naphthalene tetracarboxylic acid monoanhydride monobenzimidazole (NMM, see Figure S 4 A for structure).

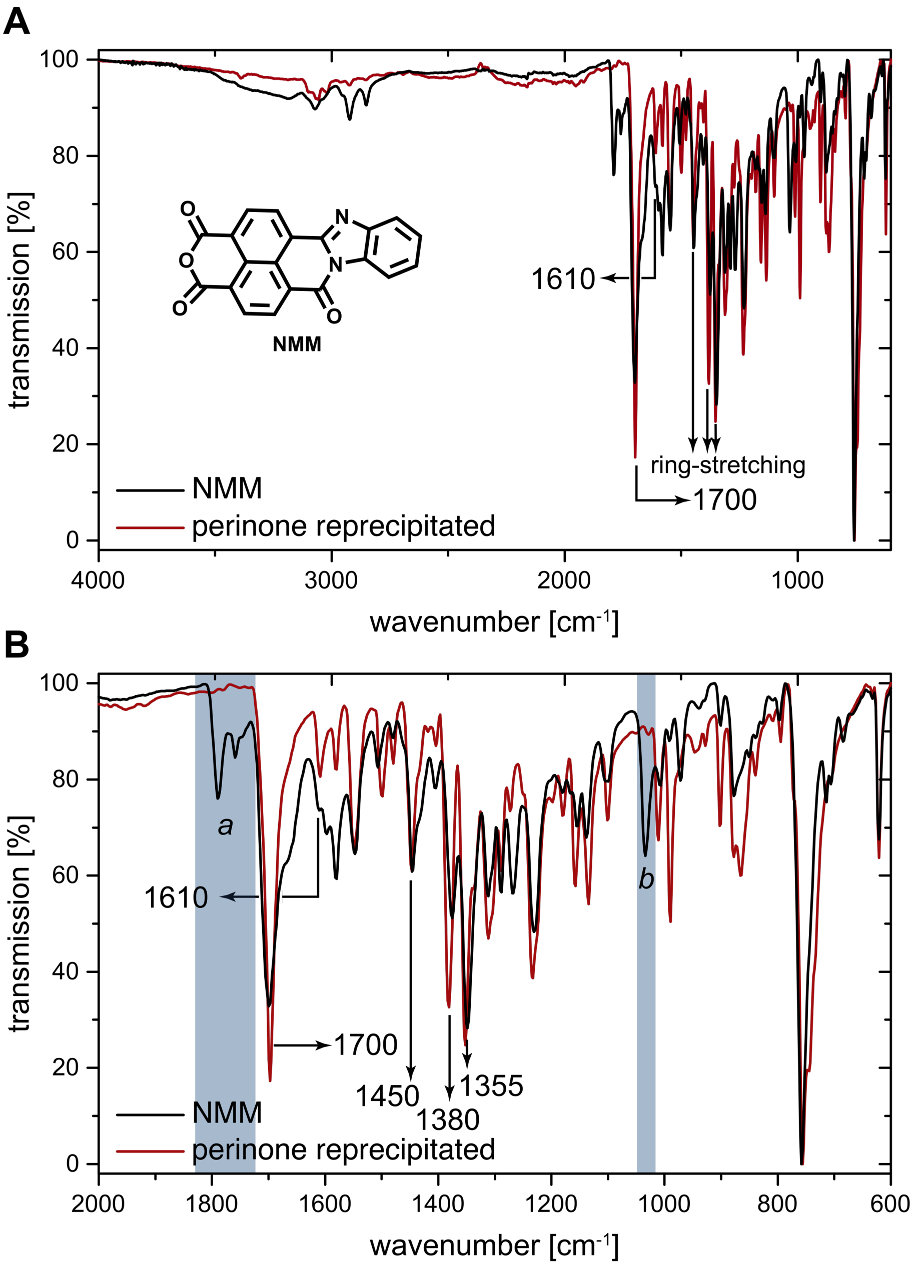

Figure S 4: Superimposed ATR-FTIR spectra of pure perinone and byproduct (A - full spectrum; B - zoom into 2000 cm^{-1} to 600 cm^{-1} region): Major differences are highlighted by blue boxes and letters *a* and *b*. Features that both spectra share are indicated with black arrows.

4.1.3 Incomplete Perinone

The ATR-FTIR spectrum of the incomplete product synthesized in a non-stirred batch autoclave at T_R = 200 °C for t_R = 4 h is depicted in Figure S 5. An anhydride C=O stretching mode at 1770 cm^{-1} with two shoulders at 1760 cm^{-1} and 1780 cm^{-1} (as in NBA; highlighted by blue box) is clearly present. Due to the intense red color of the obtained product and the presence of all characteristic perinone modes ($\nu(C{=}O)_{perinone}$ ≈ 1700 cm^{-1}, ν(C=N/C=C) ≈ 1610 cm^{-1} and the benzimidazole ring-stretching modes at 1450 cm^{-1}, 1380 cm^{-1} and 1355 cm^{-1}) we can assume that significant amounts of perinone as well as NMM have already formed. Thus, we can conclude incomplete conversion.

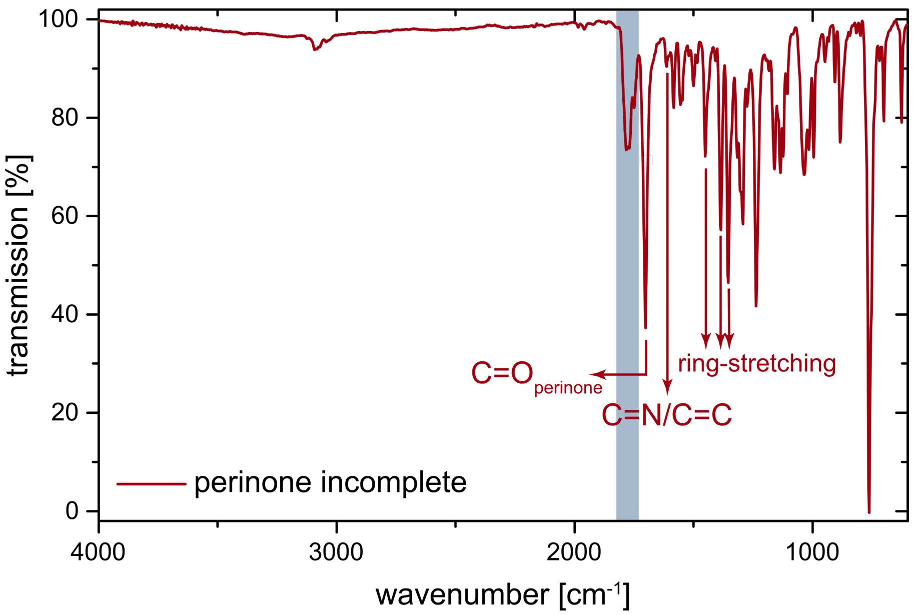

Figure S 5: ATR-FTIR spectrum of the obtained incomplete product synthesized in a non-stirred batch autoclave at T_R = 200 °C for t_R = 4 h: The presence of the anhydride C=O stretching mode at 1770 cm^{-1} (highlighted by blue box) indicates incomplete conversion of NBA. The characteristic perinone modes are indicated in red.

The FTIR-ATR spectra of all other crude samples, where the conversion of starting compounds was incomplete (see Table S 1 and Table S 2), are very similar to Figure S 5 independent of the experimental set-up used for their preparation (non-stirred batch autoclave or stirred MW-assisted batch autoclave). Depending on t_R, T_R and the corresponding reaction progress, the mode at 1770 cm^{-1} with its two shoulders at 1760 cm^{-1} and 1780 cm^{-1} is more or less intense.

4.1.4 Oxidative Polymerization Products in Aqueous Supernatant

After evaporating the orange, aqueous supernatant phase (that was always found in the glass liner after the HT reaction) to dryness a small amount of dark purple powder was obtained. The corresponding ATR-FTIR spectrum is shown in Figure S 6. Besides characteristic N-H stretching modes and other modes resulting from residual *o*-PDA (1590 cm^{-1}, 1495 cm^{-1}, 1330 cm^{-1}, 1270cm^{-1} and 1160 cm^{-1}) also various other signals are found that cannot be exactly assigned but most likely correspond to a vast mixture of oxidation products of *o*-PDA.

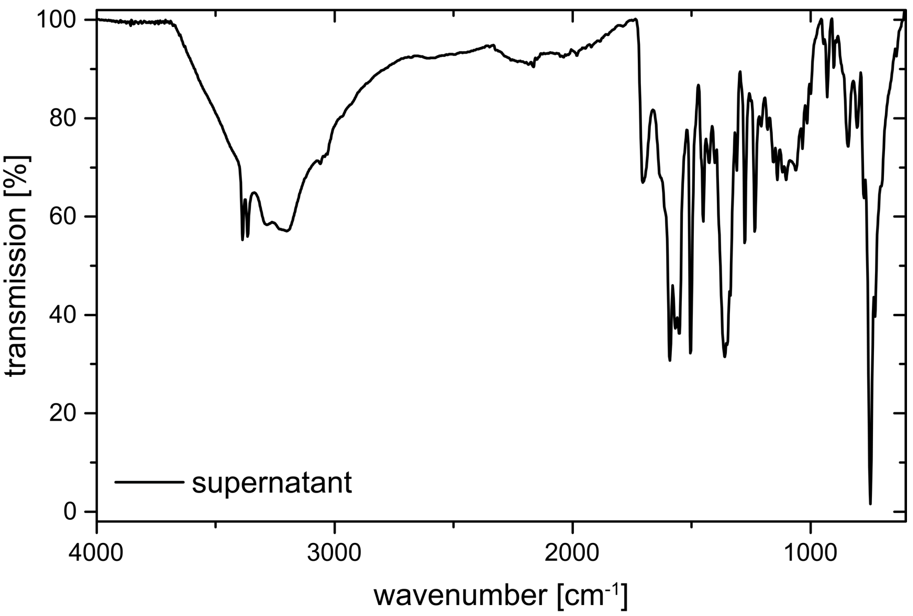

Figure S 6: ATR-FTIR spectrum of the solid residue obtained from evaporating the supernatant, aqueous phase (found in glass liner after the HT reaction) to dryness.

The longer t_R the less pronounced the N-H stretching modes get due to a proceeding oxidative polymerization of the amino compounds. However, in general, the FTIR-ATR spectra of the solid residues from the liquid supernatant are always very similar to Figure S 6.

4.1.5 Crystallized Perinone

The ATR-FTIR spectrum of crystallized perinone (reprecipitated perinone subjected to HT conditions at T_R = 250 °C, t_H = 10 min and t_R = 15 min) is shown in Figure S 7. The spectrum is completely identical to the spectrum of reprecipitated perinone (Figure S 4), which means that perinone is completely stable under HT conditions (at least for the tested t_Rs of 15 min and 60 min). The characteristic NMM modes (at 1760 cm^{-1} and 1780 cm^{-1}) cannot be found.

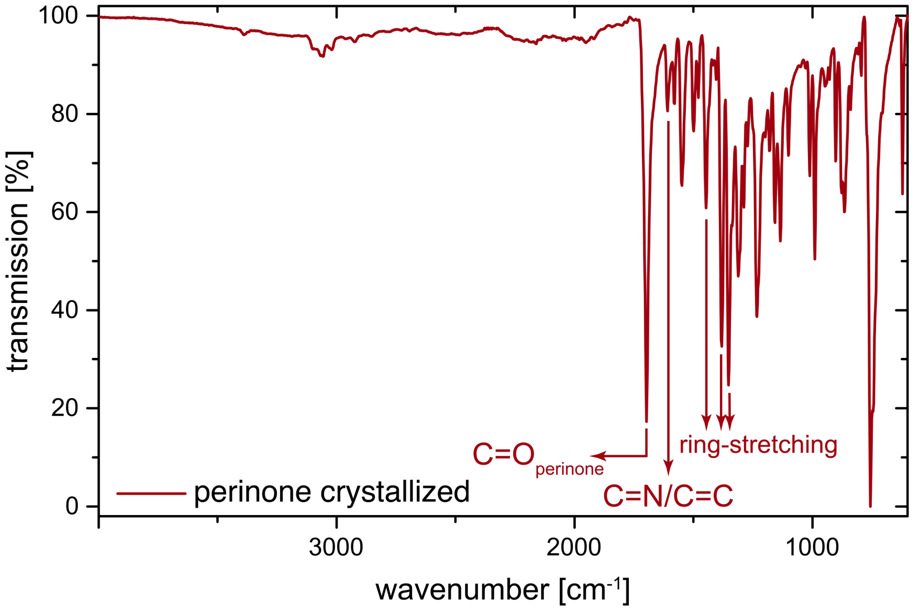

Figure S 7: ATR-FTIR spectrum of crystallized perinone (obtained after HT treatment of reprecipitated perinone at T_R = 250 °C, t_H = 10 min and t_R = 15 min): completely identical to reprecipitated perinone; characteristic NMM modes are not visible.

4.2 NMR Measurements

4.2.1 Crude Perinone

Figure S 8 A and B depict ^{1}H-NMR spectra of crude perinone (synthesized in a non-stirred batch autoclave at T_R = 200 °C for t_R = 16 h). The first spectrum (Figure S 8 A) was measured in a 10:1 deutero-chloroform ($CDCl_3$):deutero-trifluoroacetic acid (dTFA) mixture, whereas the latter one (Figure S 8 B) was recorded in a 1:1 deutero-benzene (C_6D_6):dTFA mixture.

From both spectra, it can be clearly seen that crude perinone is composed of a mixture of *cis*- and *trans*-perinone. Especially, in the spectrum recorded in C_6D_6:dTFA 1:1 (Figure S 8 B) one observes two singlets at 8.69 and 9.18 ppm that correspond to the naphthalenic protons of the *cis*-isomer H_d^c. The two doublets at 8.95 and 9.11 can be attributed to the *trans*-isomer's naphthalenic protons H_d^t. Hence, it is also possible to roughly determine the *cis*:*trans* ratio of crude perinone, which is approximately 2:3. In the spectrum recorded in $CDCl_3$:dTFA 10:1 (Figure S 8 A) the two signals for the naphthalenic protons H_d^c of the *cis*-isomer are only visible as one singlet and additionally overlap with the two doublets of the naphthalenic protons H_d^t of the *trans*-isomer.[iv]

Moreover, we observe signals for the benzimidazolic protons H_a, H_b and H_c in both spectra. In case of the C_6D_6:dTFA 1:1 spectrum the H_c protons for the two isomers are separately visible, whereas in $CDCl_3$:dTFA 10:1 they completely overlap. The other benzimidazolic protons H_a and H_b of the isomers overlap in both spectra. However, the integral values of the different protons do not exactly match the expected values. Furthermore, in addition to the intense signals for *cis*- and *trans*-perinone, also other, rather weak signals (labeled as A, B and B') are found in both spectra that do not correspond to either of the perinone isomers. Since neither the ATR-FTIR spectrum nor the ^{1}H-NMR spectrum shows any modes or signals related to unreacted *o*-PDA or NBA, we concluded that these signals must correspond to a byproduct (or a mixture of byproducts), which consequently leads to the above mentioned incorrect integral values. In further consequence, this prevents an exact determination of the *cis*:*trans*-ratio and only enables the above mentioned estimation of approximately 2:3. The combined results of various analytical techniques (ATR-FTIR, ^{1}H-NMR and LDI HRMS) finally allowed to identify the byproduct as NMM. Consequently, it was possible to determine the amount of NMM in crude perinone. The corresponding calculation is explained in subsection 4.2.8.

The ^{1}H-NMR spectra of all other crude samples, where full conversion of starting compounds was achieved (see Table S 1 and Table S 2), are completely identical to Figure S 8 independent of the experimental set-up used for their preparation (non-stirred batch autoclave or stirred MW-assisted batch autoclave).

For reasons of comparison, the ^{1}H-NMR spectra of commercially available, pure *trans*-perinone are shown in Figure S 16.

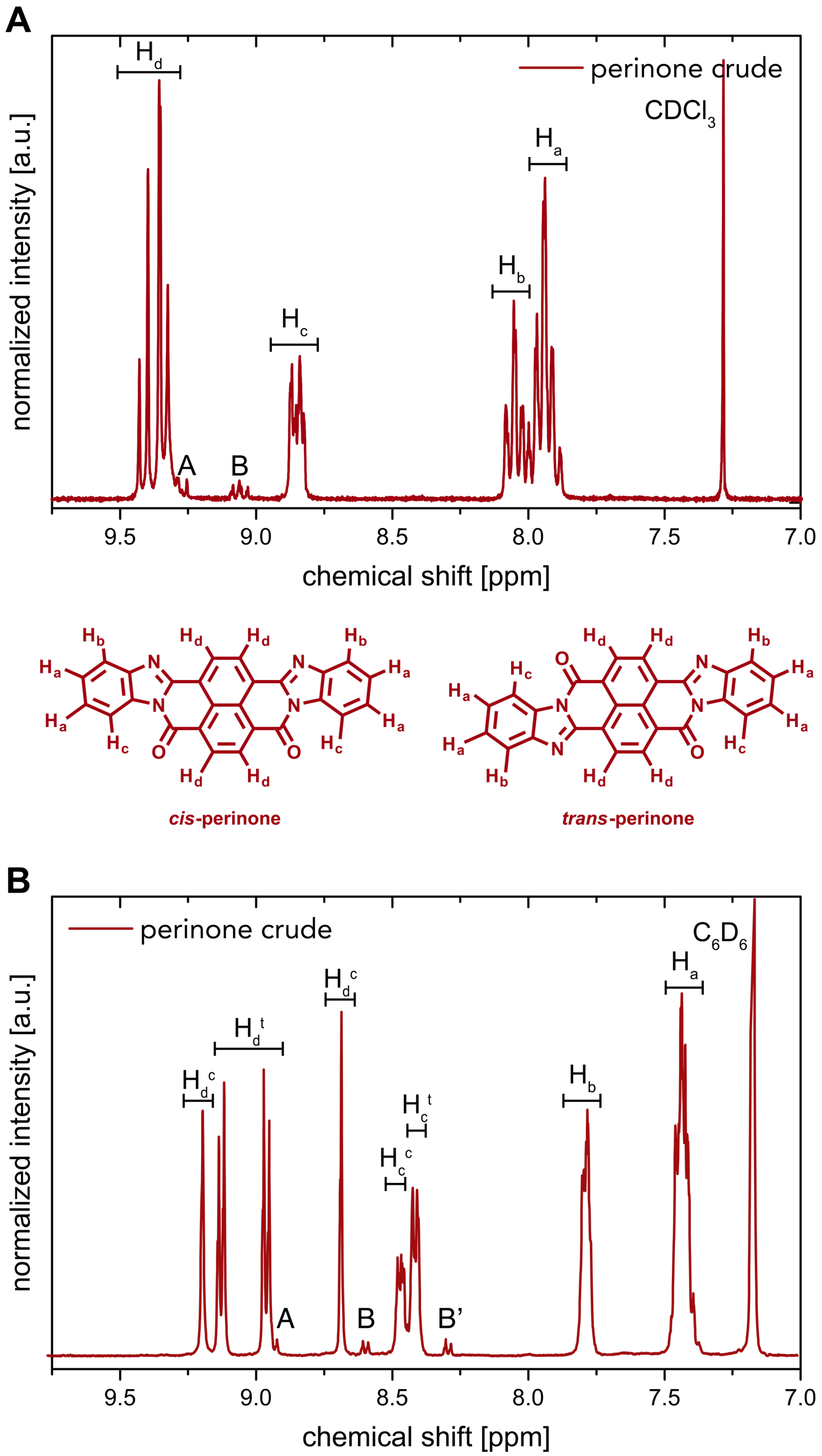

Figure S 8: ^{1}H-NMR spectra of crude perinone (synthesized in a non-stirred batch autoclave at T_R = 200 °C for t_R = 16 h) and peak assignment for H_a, H_b, H_c and H_d: A - spectrum measured in $CDCl_3$:dTFA 10:1: naphthalenic protons H_d of the isomers overlap; B - spectrum measured in C_6D_6:dTFA 1:1: differentiation between naphthalenic protons H_d of *cis*-isomer (H_d^c) and *trans*-isomer (H_d^t) is possible, which allows for determining a *cis*:*trans*-ratio of roughly 2:3; both spectra (A and B) show, that crude perinone contains a minor byproduct (signals labeld as A, B and B').

4.2.2 Byproduct

In Figure S 9 a superimposition of the ^{1}H-NMR spectra of crude perinone (synthesized in a non-stirred batch autoclave at T_R = 200 °C for t_R = 16 h) and the byproduct (NMM; separated *via* selective precipitation) measured in $CDCl_3$:dTFA 10:1 is shown. Both spectra have some signals in common, thus also confirming partial chemical similarity. The main difference are the peaks labeled as H_d and H_e.

In contrast to crude perinone's spectrum, the multiplets H_d and H_e (labeled as A and B in Figure S 8) are of major intensity now. These two signals correspond to NMM's naphthalenic unit, whereas the signlas H_a, H_b and H_c can be assigned to the benzimidazolic protons, which is completely in accordance with the spectrum measured for perinone. Furthermore, the fraction isolated as byproduct contains non-negligible amounts of *cis*-perinone (small singlet at 9.4 ppm which is labeled as P; mass confirmed *via* LDI HRMS).

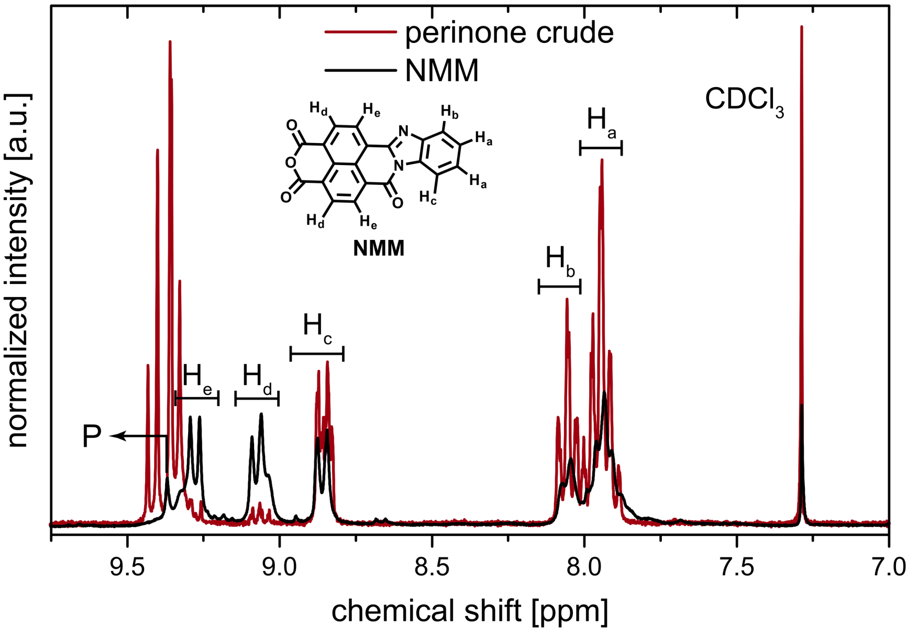

Figure S 9: Superimposed ^{1}H-NMR spectra of crude perinone (synthesized in a non-stirred batch autoclave at T_R = 200 °C for t_R = 16 h) and byproduct (separated *via* selective precipitation) measured in $CDCl_3$:dTFA 10:1: H_a, H_b, H_c, H_d and H_e indicate the characteristic NMM signals. The singlet labeled as P can be attributed to residual *cis*-perinone in the obtained byproduct fraction.

4.2.3 Reprecipitated Perinone

The ^{1}H-NMR spectra of pure perinone (*i.e.* after NMM has been removed *via* selective precipitation) in $CDCl_3$:dTFA 10:1 and C_6D_6:dTFA 1:1 (Figure S 10 A and B) look as expected and also the integral values perfectly fit ($\int H_a = 4$, $\int H_b = 2$, $\int H_c = 2$ and $\int H_d = 4$). The spectra in the different solvent mixtures are elucidated in more detail in subsection 4.2.1. The formerly present peaks labeled as A, B and B' in Figure S 8 A and B, which originated from NMM's presence (indicated with black arrows in Figure S 10) have completely vanished. Hence, it can be stated that selective precipitation from TFA with H_2O is an appropriate method for purifying hydrothermally synthesized

perinone. Now it is possible to correctly determine the *cis*:*trans*-ratio, which is exactly 2:3.

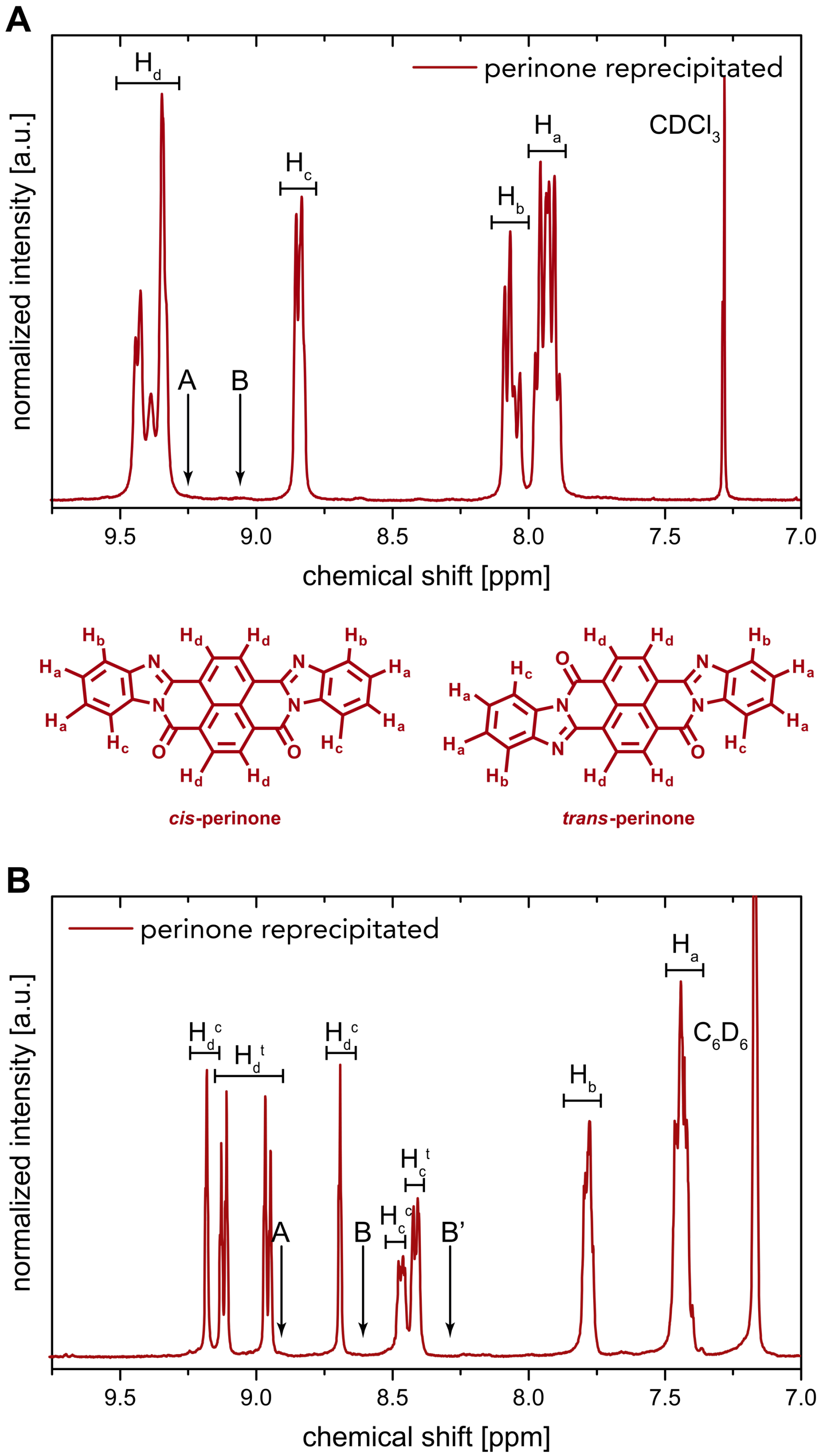

Figure S 10: ^{1}H-NMR spectra of pure perinone after selective precipitation and peak assignment for H_a, H_b, H_c and H_d. Previous position of peaks originating from NMM are indicated with black arrows and the letters A, B and B'. The integrals now match the expected values: $\int H_a = 4$, $\int H_b = 2$, $\int H_c = 2$ and $\int H_d = 4$; A - spectrum measured in $CDCl_3$:dTFA 10:1; B - spectrum measured in C_6D_6:dTFA 1:1: differentiation between naphthalenic protons H_d of *cis*-isomer (H_d^c) and *trans*-isomer (H_d^t) is possible, which allows for determining a *cis*:*trans*-ratio of 2:3.

The ^{13}C-NMR spectrum of pure perinone (Figure S 11) is in perfect accordance with the literature.[ii]

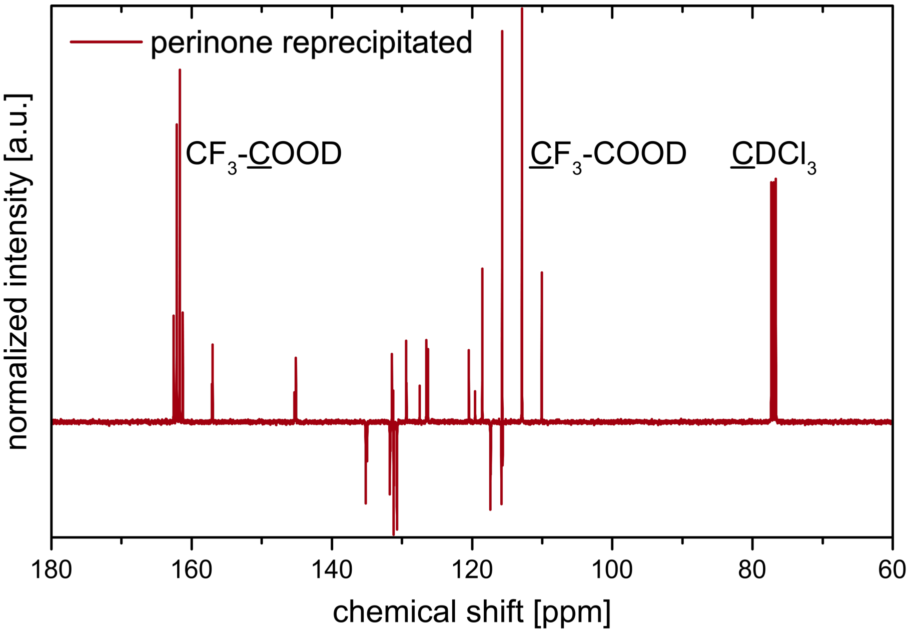

Figure S 11: ^{13}C-NMR spectrum of pure perinone after selective precipitation measured in $CDCl_3$:dTFA 10:1.

^{1}H-NMR:
δ [ppm] (400 MHz, $CDCl_3$:dTFA = 10:1, mixture of *cis*- and *trans*-perinone) = 9.27 - 9.50 (m, 4 H), 8.78 - 8.89 (m, 2 H), 7.99 - 8.12 (m, 2 H), 7.83 - 7.99 (m, 4 H).

δ [ppm] (400 MHz, C_6D_6:dTFA = 1:1, *cis*-perinone) = 9.18 (s, 2 H), 8.69 (s, 2 H), 8.43 - 8.50 (m, 2 H), 7.73 - 7.84 (m, 2 H), 7.37 - 7.51 (m, 4 H).
δ [ppm] (400 MHz, C_6D_6:dTFA = 1:1, *trans*-perinone) = 9.11 (d, *J*=7.80 Hz, 2 H) 8.95 (d, *J*=7.80 Hz, 2 H), 8.37-8.35 (m, 2 H), 7.73 - 7.84 (m, 2 H), 7.37 - 7.51 (m, 4 H).

^{13}C-NMR:
δ [ppm] (400 MHz, $CDCl_3$:dTFA = 10:1, mixture of *cis*- and *trans*-perinone = 10:1) = 156.96, 156.86, 145.19, 144.98, 135.03, 134.82, 131.59, 131.50, 131.28, 131.12, 131.06, 131.03, 130.60, 130.57, 129.26, 129.20, 127.32, 126.36, 126.17, 126.15, 120.31, 119.43, 117.24, 117.19, 115.68, 115.49.

4.2.4 Incomplete Perinone

The ^{1}H-NMR spectra of the incomplete products synthesized in a non-stirred batch autoclave at T_R = 200 °C for t_R = 2 h and 4 h, respectively, are depicted in Figure S 12. It can be clearly seen that besides NMM (peaks labeled as A and B) an additional singlet (highlighted by a blue box) is present in the spectrum, which corresponds to unreacted NBA. Furthermore, it becomes evident that the amounts of NMM and NBA decrease with increasing t_R.

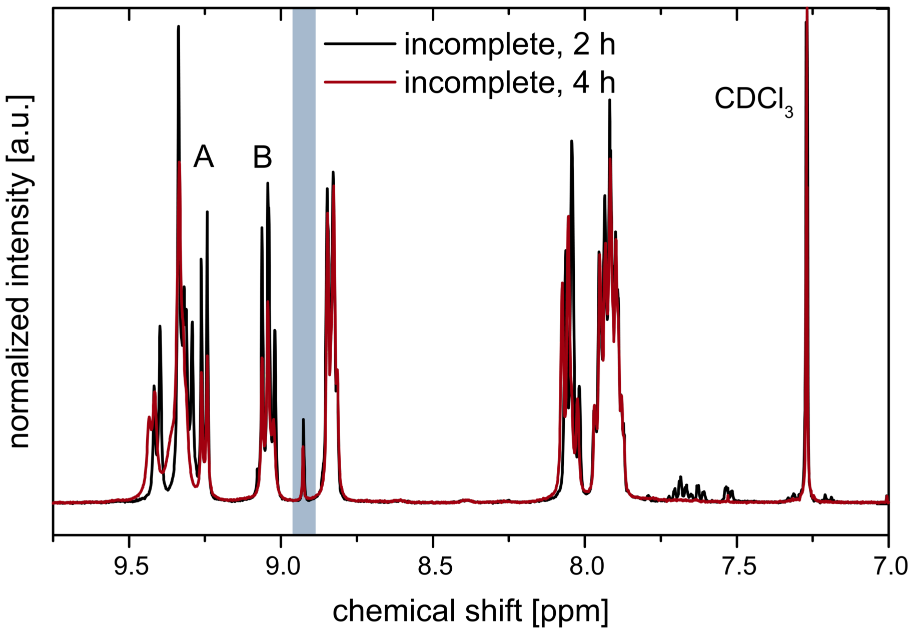

Figure S 12: ^{1}H-NMR spectra of incomplete perinone synthesized in a non-stirred batch autoclave at T_R = 200 °C for t_R = 2 h and 4 h, respectively (measured in $CDCl_3$:dTFA 10:1): The singlet, which corresponds to unreacted NBA is highlighted by a blue box. The NMM signals labeled as A and B as well as the NBA-singlet (highlighted by blue box) decrease over the course of the reaction.

The ^{1}H-NMR spectra of all other crude samples, where the conversion of starting compounds was incomplete (see Table S 1 and Table S 2), are very similar to Figure S 12 independent of the experimental set-up used for their preparation (non-stirred batch autoclave or stirred MW-assisted batch autoclave). Depending on t_R, T_R and the corresponding reaction progress the above mentioned NMM and NBA signals are more or less intense.

4.2.5 Oxidative Polymerization Products in Aqueous Supernatant

After evaporating the orange, aqueous supernatant phase (that was always found in the glass liner after the HT reaction) to dryness a small amount of dark purple powder was obtained. The corresponding ^{1}H- and ^{13}C-NMR spectra are shown in Figure S 13 and Figure S 14 , respectively. The multitude of signals in the aromatic regions of both spectra is highly indicative for oxidative polymerization of *o*-PDA.

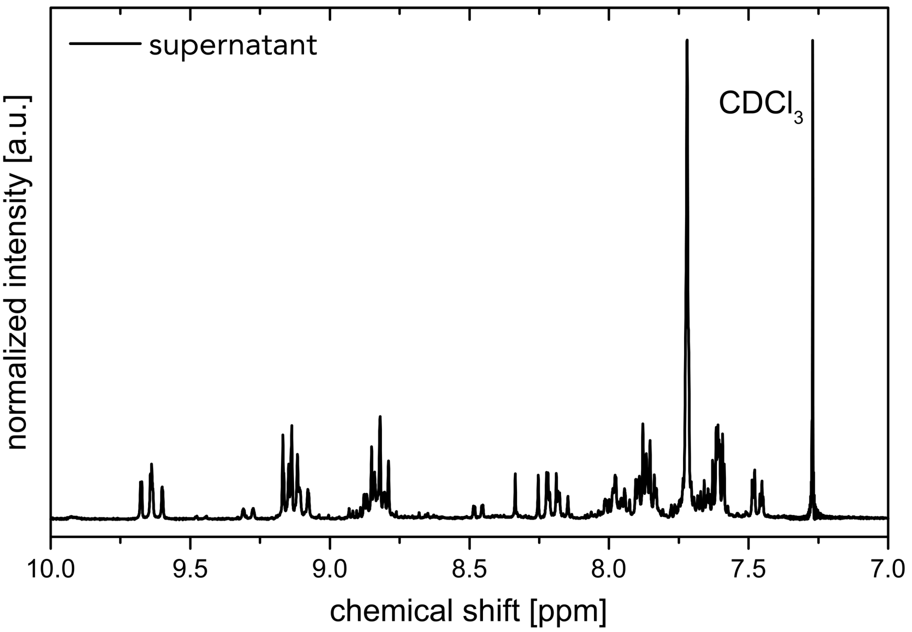

Figure S 13: ^{1}H-NMR spectrum (measured in $CDCl_3$:dTFA 10:1) of the solid residue obtained from evaporating the supernatant, aqueous phase (found in glass liner after the HT reaction) to dryness.

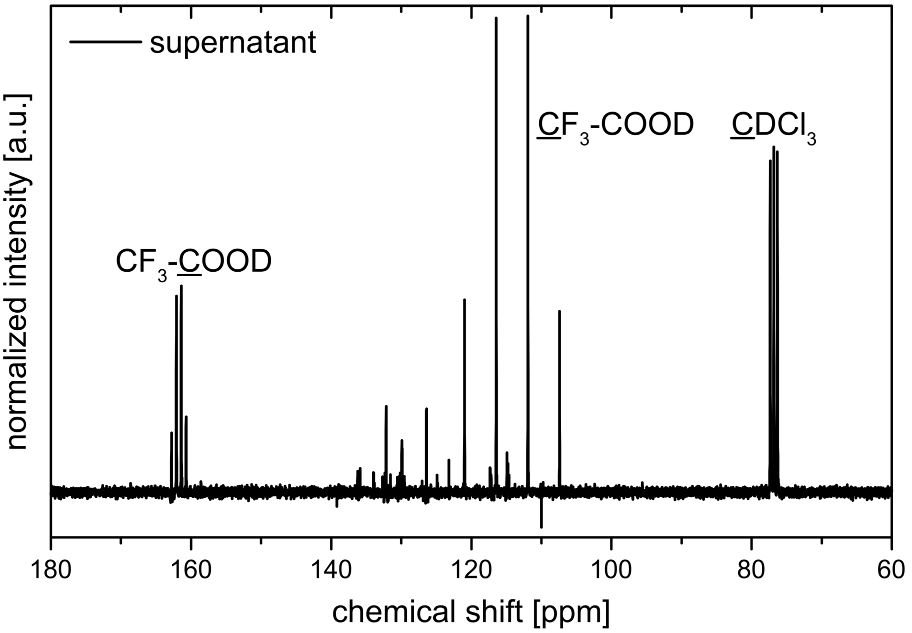

Figure S 14: ^{13}C-NMR spectrum (measured in $CDCl_3$:dTFA 10:1) of the solid residue obtained from evaporating the supernatant, aqueous phase (found in glass liner after the HT reaction) to dryness.

4.2.6 Crystallized Perinone

The ^{1}H-NMR spectra (measured in in $CDCl_3$:dTFA 10:1 and in C_6D_6:dTFA 1:1, respectively) of crystallized perinone (reprecipitated perinone subjected to HT conditions at T_R = 250 °C, t_H = 10 min and t_R = 15 min) are shown in Figure S 15. The spectra are completely identical to the ones of reprecipitated perinone (Figure S 10). The characteristic NMM signals (highlighted by A, B and B′ in Figure S 8) cannot be found. Furthermore, the *cis*:*trans*-ratio of 2:3 does not change upon crystallizing.

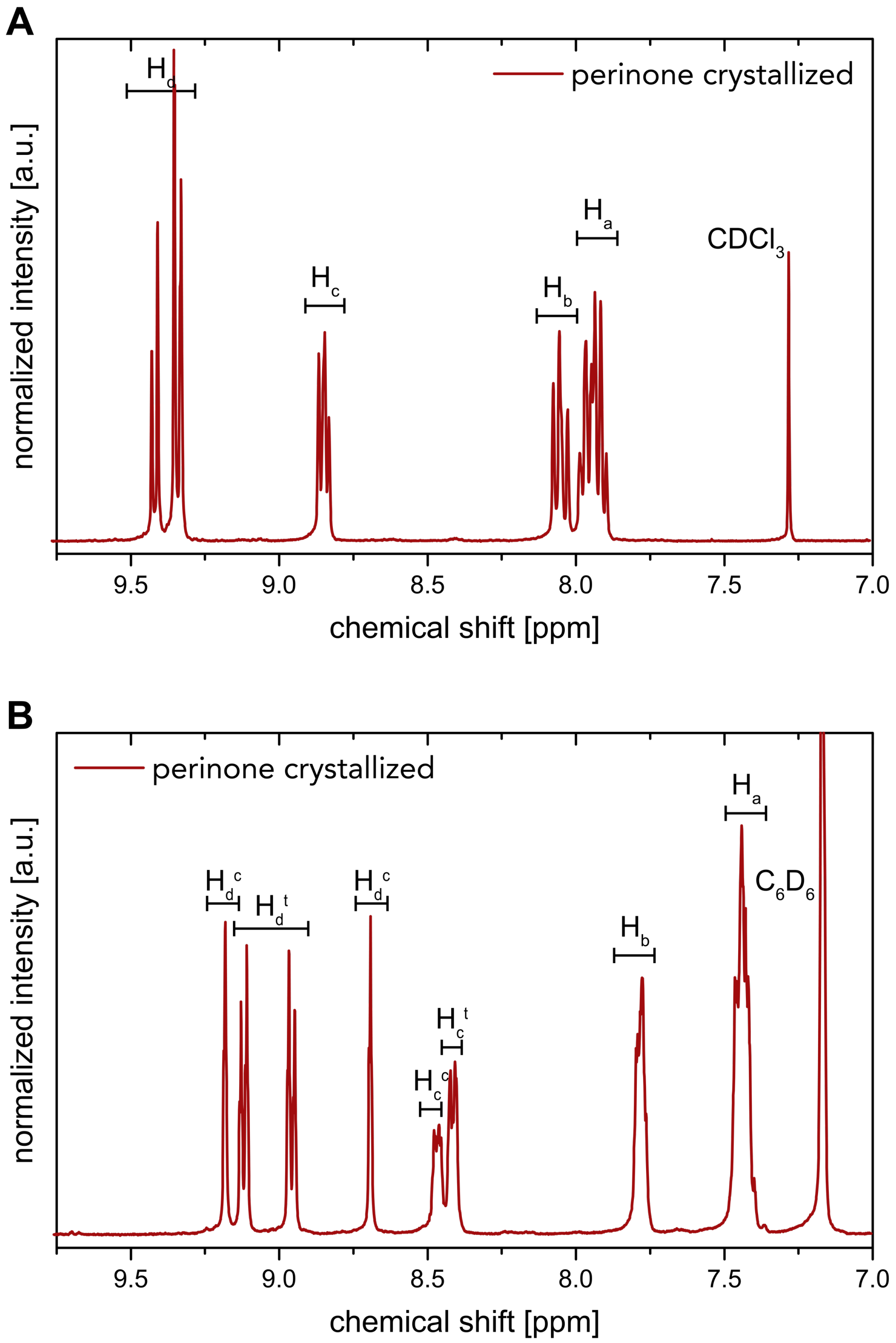

Figure S 15: ^{1}H-NMR spectra (A - in $CDCl_3$:dTFA 10:1; B - in C_6D_6:dTFA 1:1) of crystallized perinone (obtained after HT treatment of reprecipitated perinone at T_R = 250 °C, t_H = 10 min and t_R = 15 min): completely identical to reprecipitated perinone; NMM signals are not found and *cis*:*trans*-ratio (2:3) does not change upon crystallizing.

4.2.7 *trans*-Perinone

The ^{1}H-NMR spectra (measured in in $CDCl_3$:dTFA 10:1 and in C_6D_6:dTFA 1:1, respectively) of commercially available *trans*-perinone (used as received) are shown in Figure S 16. For an explanation of the assignment of H_a, H_b, H_c and H_d see subsection 4.2.1.

Especially in Figure S 16 B one can easily see that the characteristic doublets for the naphtalenic protons H_d^t at 8.95 and 9.11 ppm are present, whereas the singlets for *cis*-perinone at 8.69 and 9.18 pmm are completely missing.

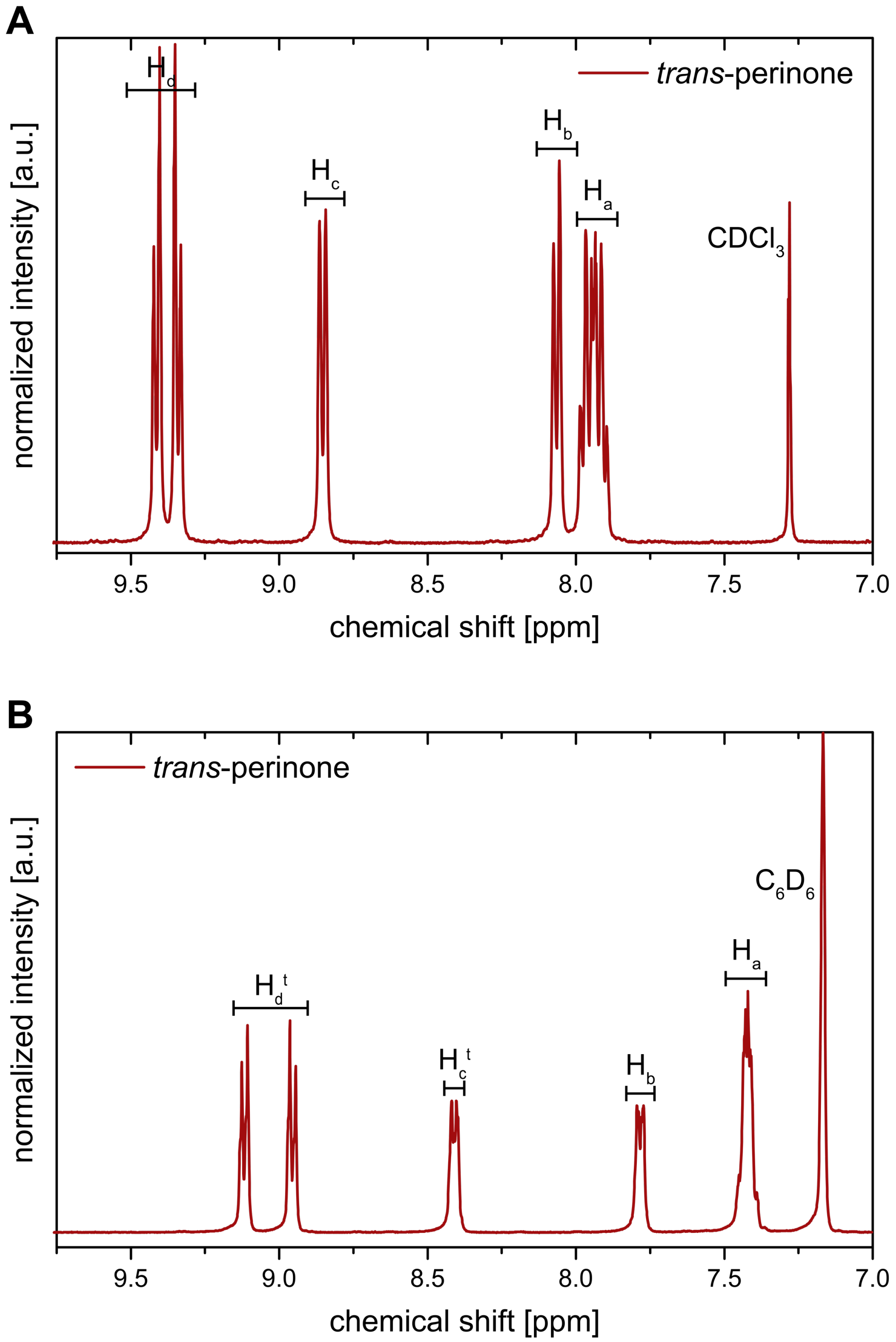

Figure S 16: ^{1}H-NMR spectra (A - in $CDCl_3$:dTFA 10:1; B - in C_6D_6:dTFA 1:1) of commercially available *trans*-perinone: characteristic doublets for *trans*-perinone are present, whereas the singlets for *cis*-perinone are completely missing.

The ^{1}H-NMR spectra of *trans*-perinone after HT crystallization (at T_R = 250 °C, t_H = 10 min and t_R = 15 min or 60 min) are completely identical to the ones measured before HT treatment (depicted in Figure S 16). Since neither changes in the spectra nor in the macroscopic appearance occur, we assume that *trans*-perinone is stable under HT conditions (at least for the examined t_Rs of 15 and 60 min, respectively).

4.2.8 Determining the Byproduct Content in Crude Perinone

The NMM content in crude perinone (synthesized in a non-stirred batch autoclave at T_R = 200 °C for t_R = 16 h) was determined from the ^{1}H-NMR spectrum measured in $CDCl_3$:dTFA 10:1. The peaks, which were taken into account are highlighted in Figure S 17 by blue boxes and labeled as X and Y. X corresponds to 4 Hs of perinone (*cis*- and *trans*-isomers) and 2 Hs of NMM, whereas Y only corresponds to 2 Hs of NMM. Hence, it is possible to calculate NMM's concentration (in mol%) in crude perinone using the following equations:

$$X = 2\,n + 4\,p \quad and \quad Y = 2\,n$$

$$n = \frac{Y}{2} \quad and \quad p = \frac{X - Y}{4}$$

$$mol\%\;of\;NMM = \frac{n}{n + p} = \frac{2\,Y}{X + Y}$$

where:

X, Y … values of the appropriate integrals labeled in Figure S 17
n… mole of NMM
p… mole of perinone

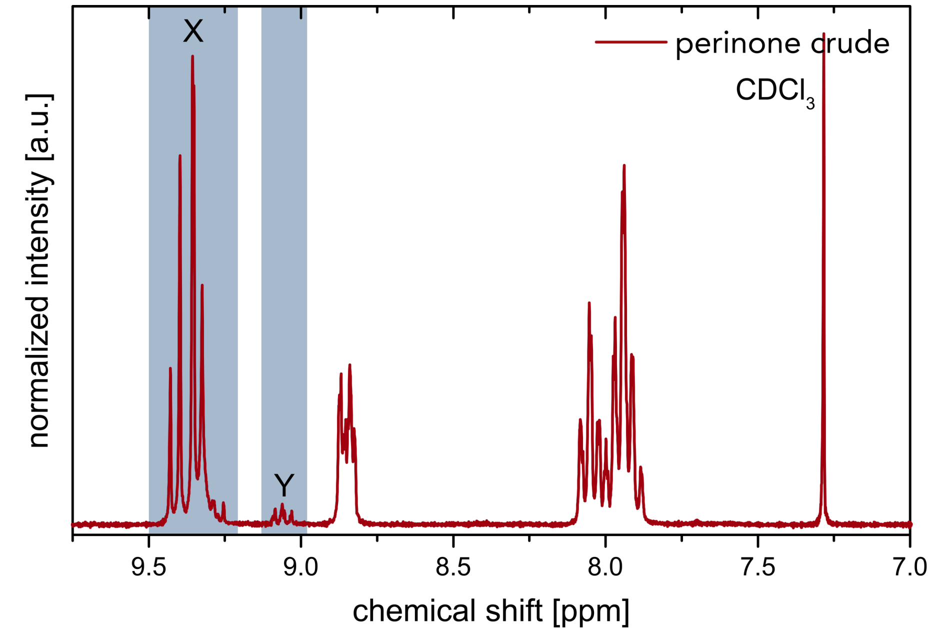

Figure S 17: Illustration of the NMM content determination in crude perinone (synthesized in a non-stirred batch autoclave at T_R = 200 °C for t_R = 16 h) *via* ^{1}H-NMR measured in $CDCl_3$:dTFA 10:1: relevant signals are highlighted by blue boxes and labeled as X and Y.

According to these equations a NMM content of 7 mol% with respect to the total amount of crude product was determined. This value remained approximately constant for all other samples, where full conversion of starting compounds was observed (see Table S 1 and Table S 2).

4.3 LDI HRMS Measurements

According to LDI HRMS measurements (Figure S 18) the isolated byproduct fraction (obtained *via* selective precipitation) contains not only NMM (m/z calculated: 341.06; found: 341.06), but also perinone (m/z calculated: 413.10; found: 413.11). We assume that only *cis*-perinone is present, but no *trans*-isomer due to the very characteristic singlet in the byproduct's ^{1}H-NMR spectrum (see Figure S 9).

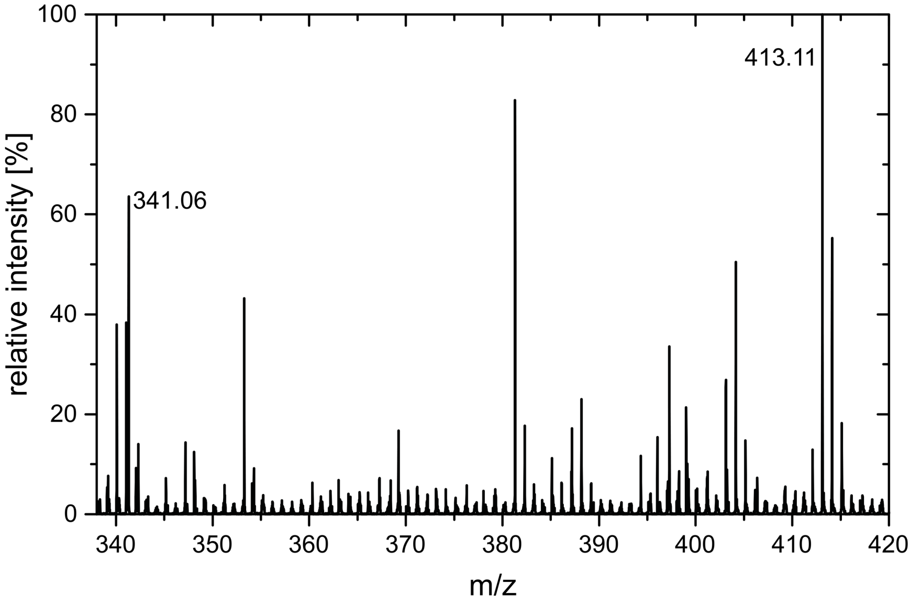

Figure S 18: LDI HRMS spectrum of the isolated byproduct fraction (obtained after selective precipitation): the isolated byproduct fraction contains NMM (m/z 341.06) and *cis*-perinone (m/z 413.11)

4.4 PXRD Measurements

4.4.1 Crude Perinone

The PXRD pattern of crude perinone (synthesized in a non-stirred batch autoclave at T_R = 200 °C for t_R = 16 h) as well as a simulated pattern derived from the crystal structure of a solid solution of *cis*- and *trans*-perinone are shown in Figure S 19. Hydrothermally synthesized crude perinone is highly crystalline and occurs as a solid solution of *cis*- and *trans*-perinone. All major reflections are found and are perfectly in accordance with the literature.[v]

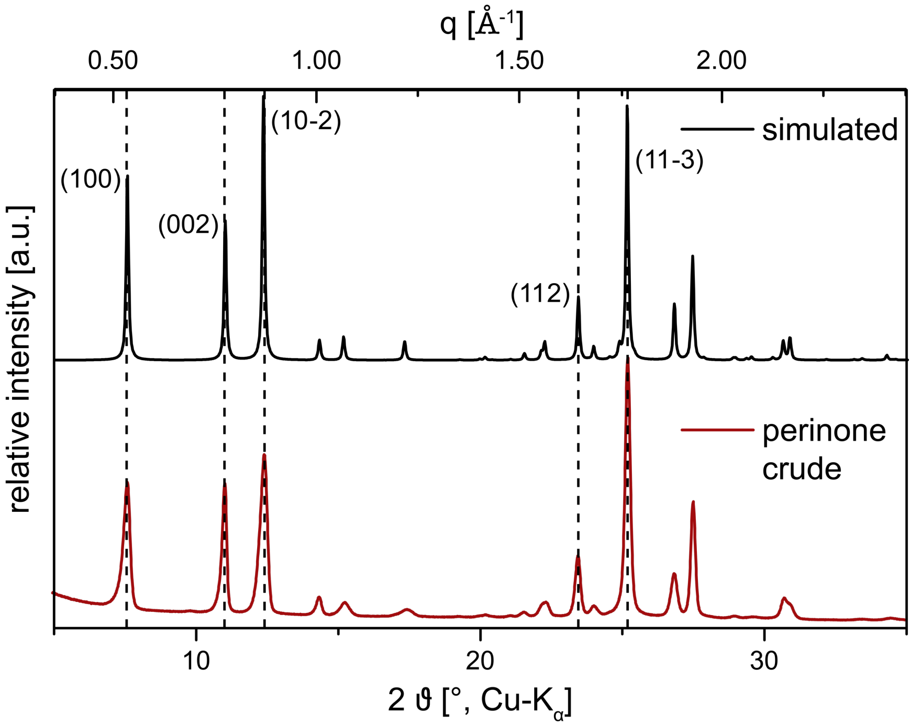

Figure S 19: PXRD pattern of crude perinone (bottom; synthesized in a non-stirred batch autoclave at T_R = 200 °C for t_R = 16 h) and a simulated pattern (top; with major reflections indicated) calculated using the crystal structure of a solid solution of *cis*- and *trans*-perinone from Ref.[v]: crude perinone is highly crystalline and the observed reflections are in perfect agreement with the previously reported pattern of the solid solution.

4.4.2 Reprecipitated Perinone

PXRD measurements revealed that selective precipitation gives rise to a solid that can be described as semi-crystalline at best, whereas crude perinone (before selective precipitation) is highly crystalline (see Figure S 20). The diffractograms of reprecipitated perinone shows 15 reflections (labeled as 1-15 in Figure S 20) between 5 and 35° (2 θ, Cu-K$_\alpha$). All reflections are much broader than the reflections in the PXRD pattern of crude perinone (solid solution). Some reflections seem to fit the pattern of crude perinone reasonably well, specifically 1, 4, 5, 10, 11, 12 and 15, however they are off-set; mostly by more than 1° (2 θ, Cu-K$_\alpha$). Moreover, the peaks that do not fit at all (2, 3, 6 and 13), are those with the highest intensities.

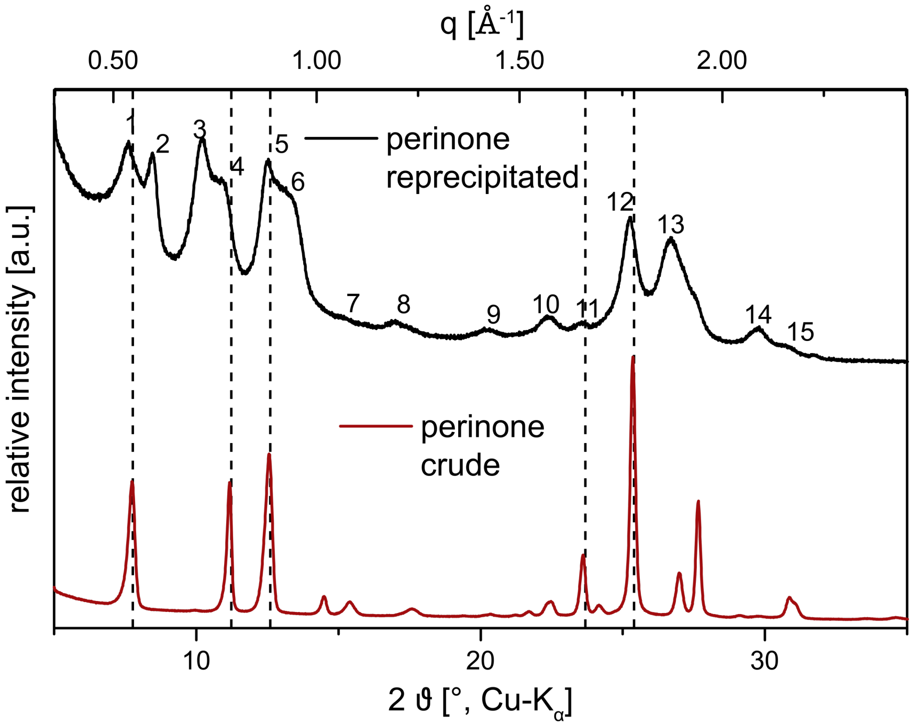

Figure S 20: PXRD patterns of crude (bottom; synthesized in a non-stirred batch autoclave at T_R = 200 °C for t_R = 16 h) and reprecipitated (top) perinone: the selective precipitation procedure significantly decreases crystallinity.

First, we suspected that the diffractogram might be an overlay of *cis*- and *trans*-isomers, resulting from a heterogeneous mixture of very small particles (hence the broad reflections) of separated, pure isomers. This hypothesis seemed reasonable, since reprecipitation is a well-known technique for fractionating *e.g.* polymers by solubility differences arising from different molecular weights. However, the diffractogram of reprecipitated perinone does also not fit either of the pure isomers (see Figure S 21).

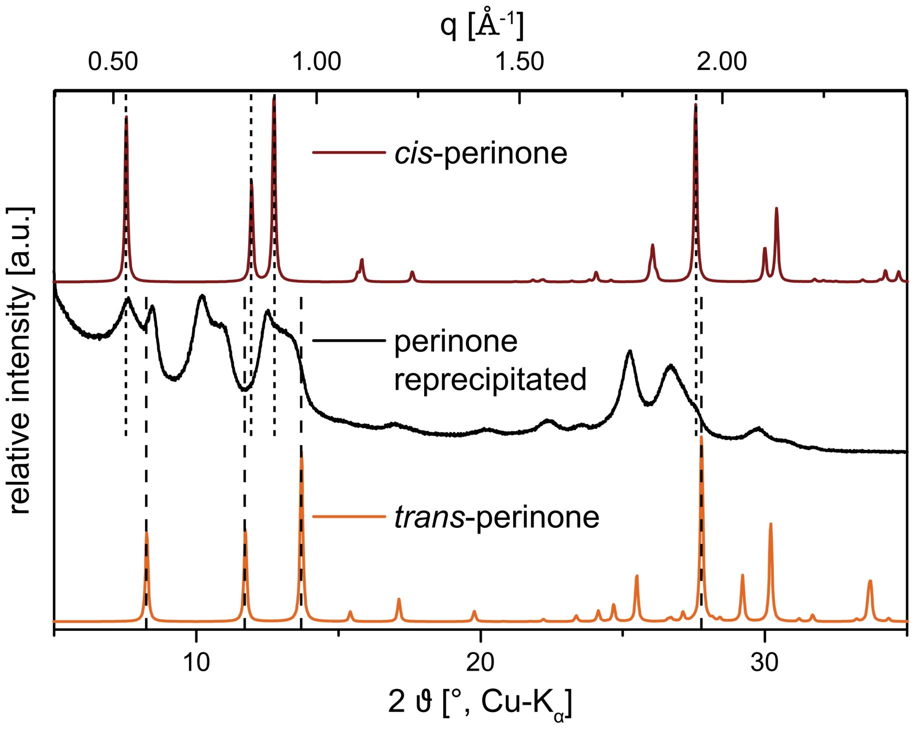

Figure S 21: PXRD pattern of reprecipitated perinone (middle) and simulated patterns for *cis*- (top) and *trans*-perinone (bottom): It can be clearly seen that reprecipitated perinone is not composed of a heterogeneous mixture of the two separated isomers as that would generate a pattern sharing all reflections of both isomers (sum pattern). *cis*- and *trans*-patterns were calculated from the crystal structures from Ref.[vi]

Given that the diffractogram evinces considerable order nonetheless, we moved on to a second hypothesis: The reprecipitation with a non-solvent rapidly quenches the perinone solution, *i.e.* the repricipitated perinone shows much more disorder than crude perinone in the crystalline state, rather comparable to a liquid crystalline (LC) phase. Considering perinone's molecular geometry (which does not significantly change upon protonation by TFA), it is conceivable that perinone also forms a LC phase: Both *cis*- and *trans*- perinone can be considered discotic mesogens. In fact, both the pure isomers and the solid solution crystallize in $P2_1/c$, and exhibit infinite offset face-to-face (OFF) π-stacks in the solid state, which can be considered tilted columns in a columnar LC phase. In further consequence, this leads to ellipsoidal column cross sections. Hence, we would expect perinone in an LC phase to occur as a columnar rectangular mesophase (Col_r). Col_r phases typically assume $P2_1/a$, $P2/a$ or $C2/m$ space groups.[vii] We attempted to index the reflections to a rectangular lattice, and all peaks fit relatively well (see Figure S 22), which is why we believe that reprecipitated perinone is indeed well described as highly disordered Col_r phase.

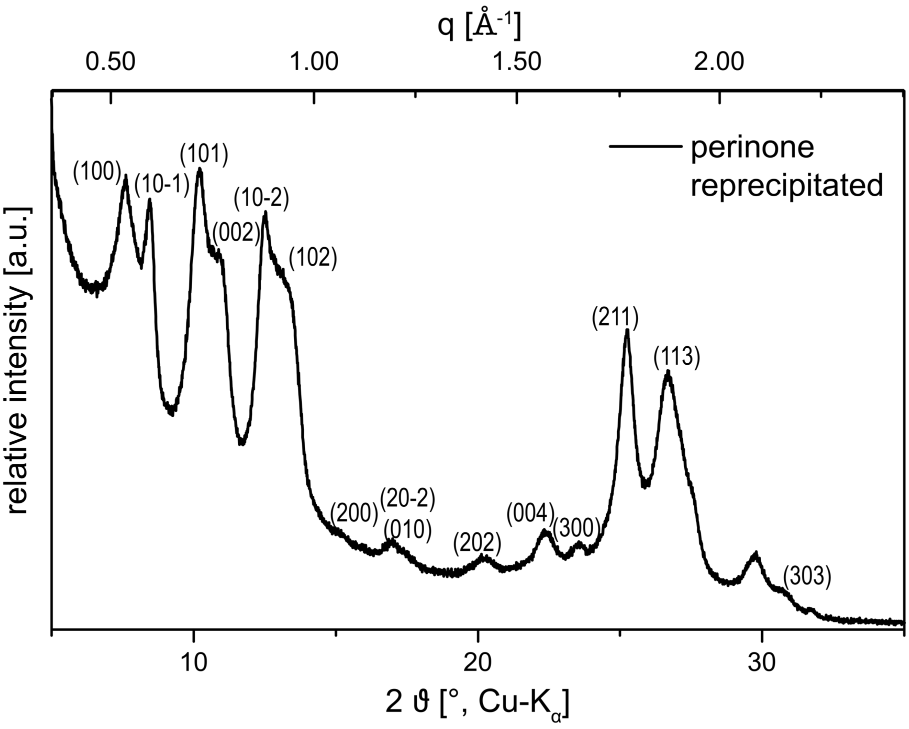

Figure S 22: PXRD pattern of reprecipitated perinone and major reflections indexed to a rectangular lattice in space group $P2_1/a$.

4.4.3 Crystallized Perinone

The PXRD patterns of crude (synthesized in a non-stirred batch autoclave at T_R = 200 °C for t_R = 16 h), reprecipitated and crystallized perinone (obtained *via* HT treatment of reprecipitated perinone at T_R = 250 °C, t_H = 10 min and t_R = 15 min) are shown in Figure S 23. Subjecting reprecipitated perinone (Col_r LC phase; see Figure S 22) to HT conditions significantly increases crystallinity of the product and gives rise to the same solid solution pattern, that was already observed prior to selective precipitation for crude perinone (Figure S 19). Hence, one can state that perinone can indeed be rapidly crystallized hydrothermally.

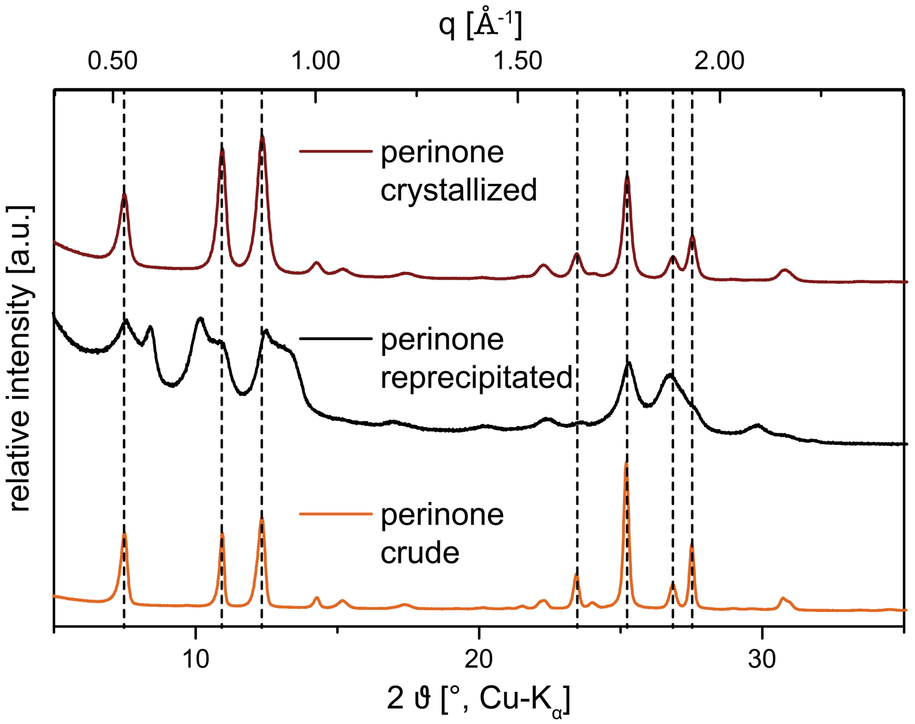

Figure S 23: PXRD patterns of crude (bottom; synthesized in a non-stirred batch autoclave at T_R = 200 °C for t_R = 16 h), reprecipitated (middle) and crystallized perinone (top; obtained *via* HT treatment of reprecipitated perinone at T_R = 250 °C, t_H = 10 min and t_R = 15 min): crude perinone is highly crystalline, whereas the purified, reprecipitated one is only semi-crystalline. Subsequent HT treatment leads to a highly crystalline product again, which proves that a rapid HT crystallization of perinone is possible.

Extending t_R of the crystallization to 60 min leads to slightly sharper reflections (compared to the sample prepared at a t_R of 15 min; shown in Figure S 23), which have the same full width at half maximum as crude perinone.

4.4.4 *trans*-Perinone

The PXRD patterns of commercially purchased *trans*-perinone before (as received) and after crystallization are shown in Figure S 24. Both patterns show the expected reflections.[vi] Furthermore, HT treatment (at T_R = 250 °C, t_H = 10 min and t_R = 15 min) gives rise to slightly sharper and more pronounced reflections, which is why we assume that a crystallization indeed took place.

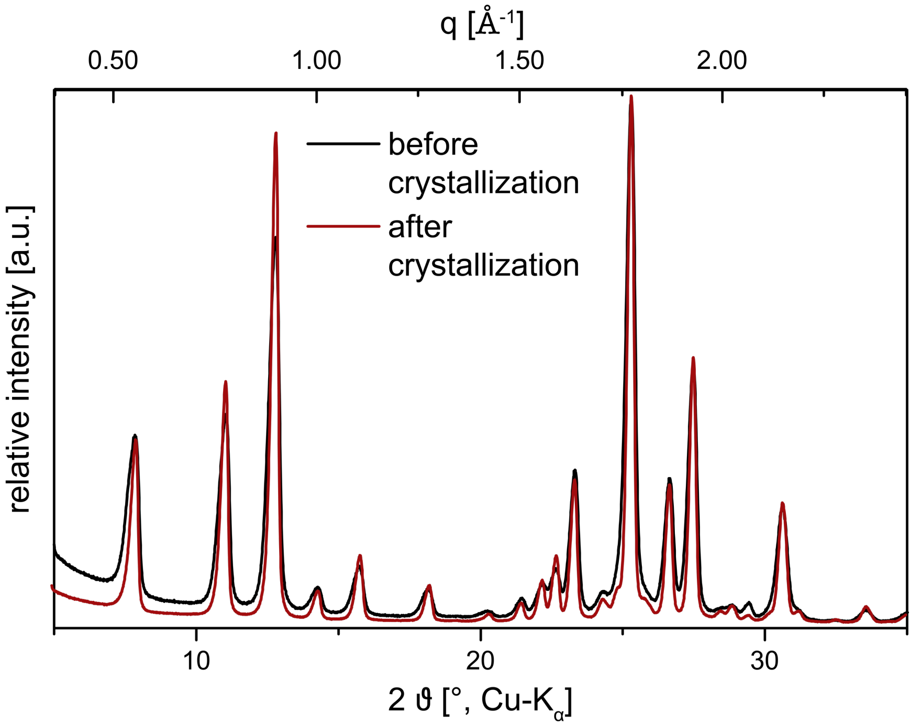

Figure S 24: PXRD patterns of *trans*-perinone before (black) and after (red) crystallization: HT treatment (at T_R = 250 °C, t_H = 10 min and t_R = 15 min) leads to slightly sharper and more pronounced reflections, which is a strong indication that *trans*-perinone can be crystallized hydrothermally.

4.5 SEM Measurements

4.5.1 Crude Perinone

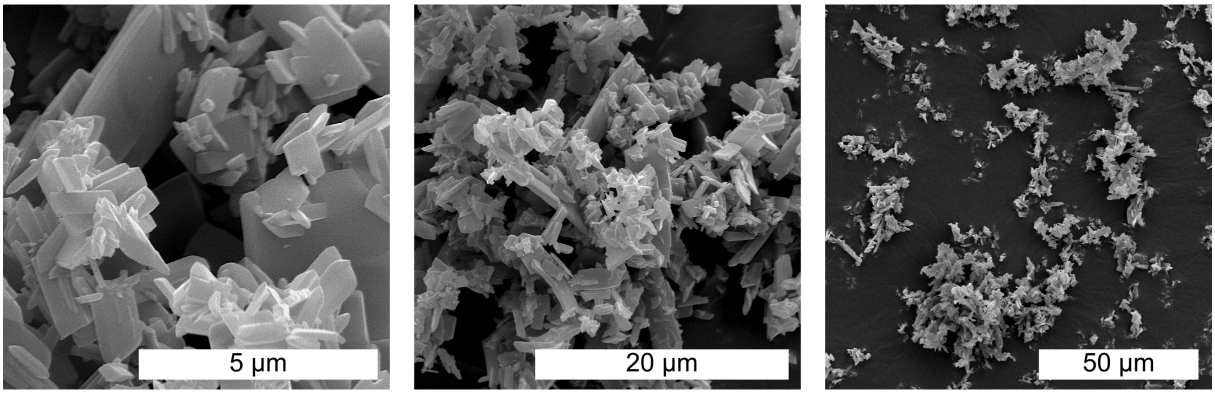

Figure S 25: SEM micrographs of crude perinone (synthesized in a stirred MW-assisted batch autoclave at T_R = 250 °C, t_H = 10 min and t_R = 2 min): crude perinone forms platelates in the size range of 0.5 - 10 µm that are partially intergrown and heavily agglomerated.

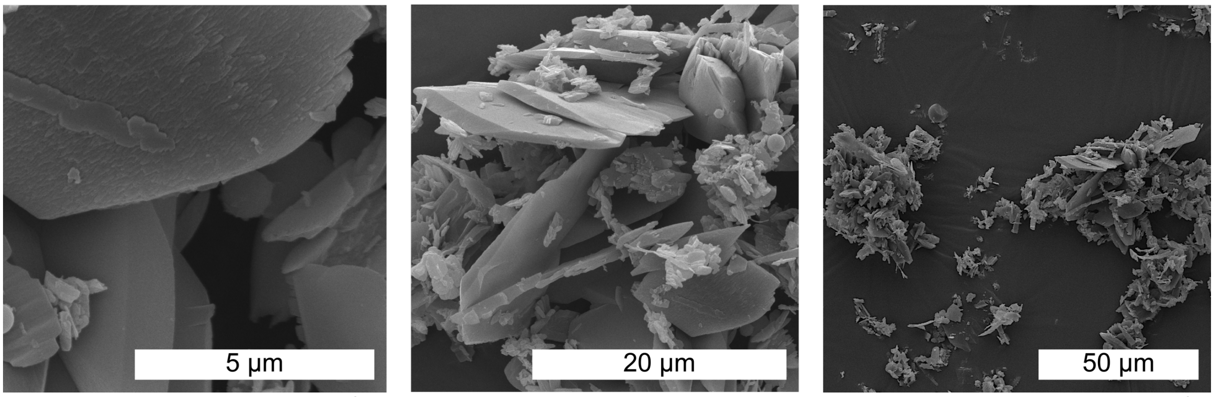

Figure S 26: SEM micrographs of crude perinone (synthesized in a non-stirred batch autoclave at T_R = 200 °C for t_R = 16 h): crude perinone forms platelates in the size range of 2 - 10 µm that are partially intergrown and heavily agglomerated.

Crude Perinone is composed of a mixture of rather flat, angular platelets of rather broad size distribution. Depending of the experimental set-up (non-stirred batch autoclave or stirred MW-assisted batch autoclave) different particle sizes are obtained. In the stirred MW-assisted batch autoclave (at T_R = 250 °C, at t_H = 10 min and t_R = 2 min) particles are typically in the size range of 2 - 10 µm (Figure S 25). In contrast, if perinone is synthesized in a non-stirred batch autoclave (at T_R = 200 °C for t_R = 16 h) typically larger platelates of 5 - 20 µm are obtained. In both cases these platelates are sometimes intergrown. Furthermore, they are mostly agglomerated into bigger structures.

The SEM micrographs of all other crude samples, where full conversion of starting compounds was achieved (see Table S 1 and Table S 2), are very similar to Figure S 25 or Figure S 26, respectively, depending on the experimental set-up (non-stirred batch autoclave or stirred MW-assisted batch autoclave).

4.5.2 Reprecipitated Perinone

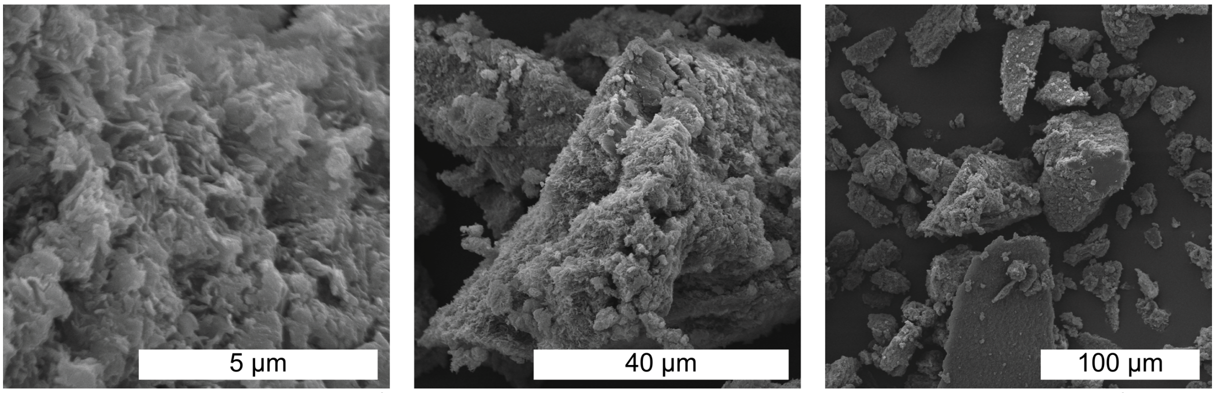

Figure S 27: SEM micrographs of reprecipitated perinone: reprecipitated perinone is composed of heavily agglomerated microparticles (0.1 - 0.5 µm) of random shape with a disordered surface texture.

After reprecipitation the morphology has completely changed. Instead of small particles in the range of 2 - 20 µm (present before reprecipitation, see Figure S 25 and Figure S 26), we observe heavily agglomerated, roundish microparticles (0.1 - 0.5 µm) with a rough random surface. These particles agglomerate into bigger structures of 20 - 200 µm.

4.5.3 Crystallized Perinone

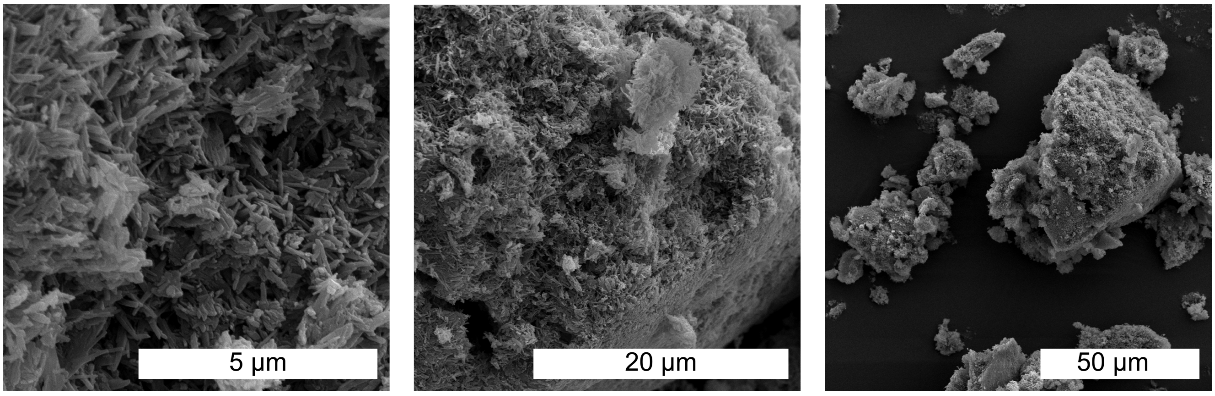

Figure S 28: SEM micrographs of crystallized perinone (obtained *via* HT treatment of reprecipitated perinone at T_R = 250 °C, t_H = 10 min and t_R = 15 min): crystallized perinone forms agglomerates (10 - 50 µm) of very fine, elongated needles (1 - 2 µm in length).

Crystallization of reprecipitated perinone (at T_R = 250 min, t_H = 10 min and t_R = 15 min) again leads to a significant change in morphology. Crystallized perinone forms very fine needles (Figure S 28), that are heavily agglomerated into bigger structures (10 - 50 µm). The needles are around 1 - 2 µm in length and partially intergrown. Extending t_R from 15 min to 60 min does not have a significant influence on the observed morphology.

4.5.3 *trans*-Perinone Before Crystallization

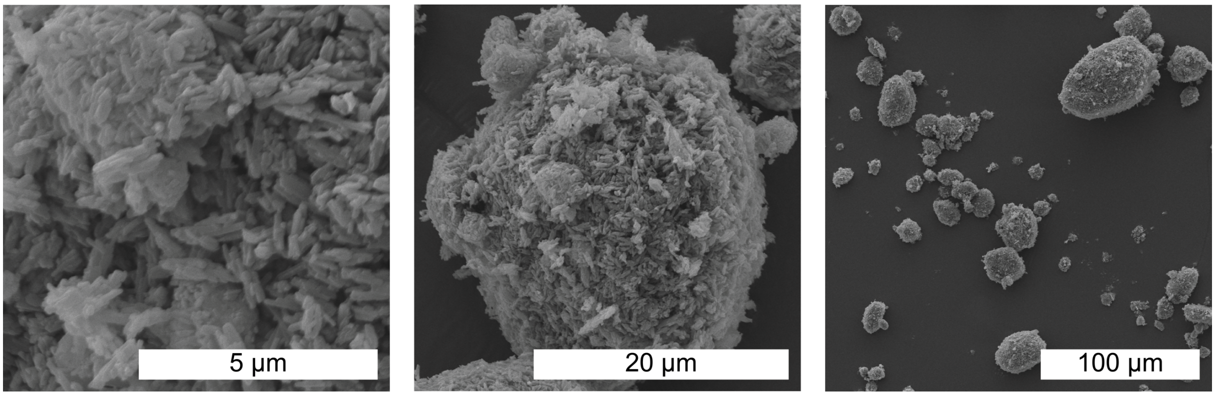

Figure S 29: SEM micrographs of commercially purchased *trans*-perinone before HT treatment: *trans*-perinone is composed of small, short needle-like particles (0.5 - 2 µm in length) that are agglomerated into roundish particles of 10 - 200 µm.

The SEM micrographs of commercially purchased *trans*-perinone (as received, *i.e.* before HT treatment; Figure S 29) show short, poorly pronounce needles, which are between 0.5 µm and 2 µm in length. These small particles agglomerate into almost spherical structures with a broad size distribution (10 - 100 µm).

4.5.6 *trans*-Perinone After Crystallization

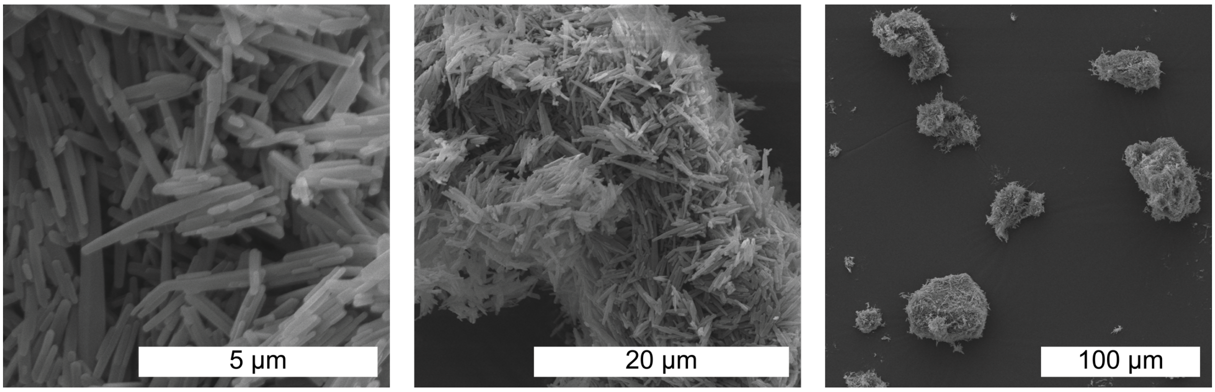

Figure S 30: SEM micrographs of commercially purchased *trans*-perinone after HT treatment: HT crystallization (at T_R = 250 °C, t_H = 10 min and t_R = 15 min) of *trans*-perinone leads to agglomerates of nicely pronounced needles (around 5 µm in length).

HT treatment (at T_R = 250 °C, t_H = 10 min and t_R = 15 min) of commercially purchased *trans*-perinone significantly improves the morphology. In the corresponding SEM micrographs (Figure S 30) we observe agglomerates (10 - 100 µm) of well pronounced needles. These needles are mainly around 5 µm in length and significantly more pronounced than before crystallization. In contrast to the sample prior to HT treatment, here we do not observe perfectly roundish shapes for the agglomerates.

5 Crystallization of Indigo and Pentacenetetrone

In order to attempt the HT crystallization of other fully conjugated, aromatic compounds we performed a set of experiments with indigo and pentacenetetrone. We chose these compounds because (*i*) they have melting points significantly above 250 °C, (*ii*) they are completely insoluble in H_2O under ambient conditions and (*iii*) they are similar as perinone interesting candidates for applications in organic electronics.

For all crystallization experiments with indigo and pentacenetetrone the optimized reaction conditions (T_R = 250 °C, t_H = 10 min and t_R = 15 min) found for the crystallization of reprecipitated perinone were applied.

The ATR-FTIR spectra before and after HT treatment (at T_R = 250 °C, t_H = 10 min and t_R = 15 min) of indigo and pentacenetetrone, respectively, are depicted in Figure S 31 and Figure S 32. As can be clearly seen, HT treatment does not lead to any changes in these spectra. This indicates that indigo and pentacenetetrone are stable (at least for the tested t_Rs of 15 min) under HT conditions. Decomposition or degradation does not occur.

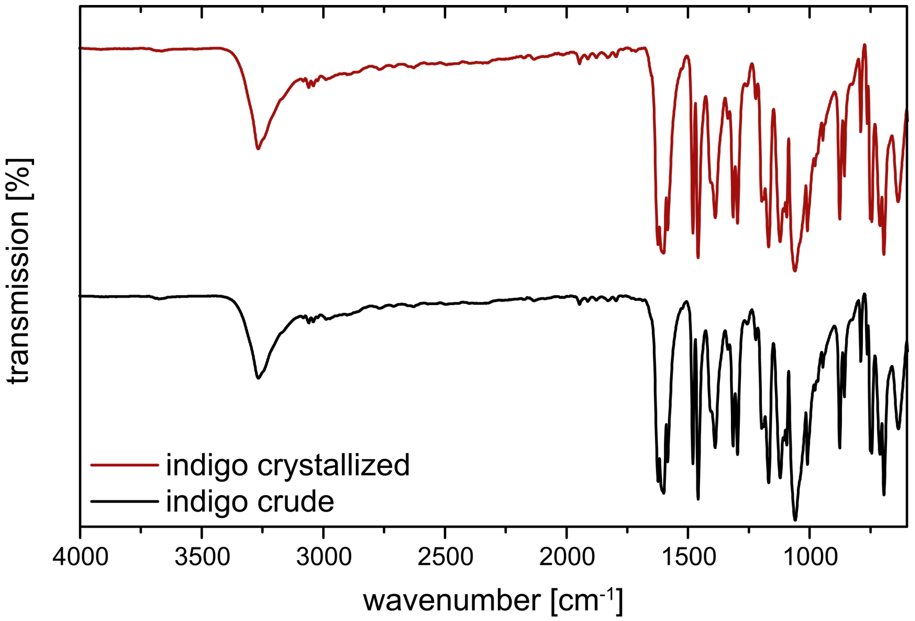

Figure S 31: ATR-FTIR spectra of indigo before and after HT treatment (at T_R = 250 °C, t_H = 10 min and t_R = 15 min): both spectra are identical, which indicates that indigo is stable under HT conditions.

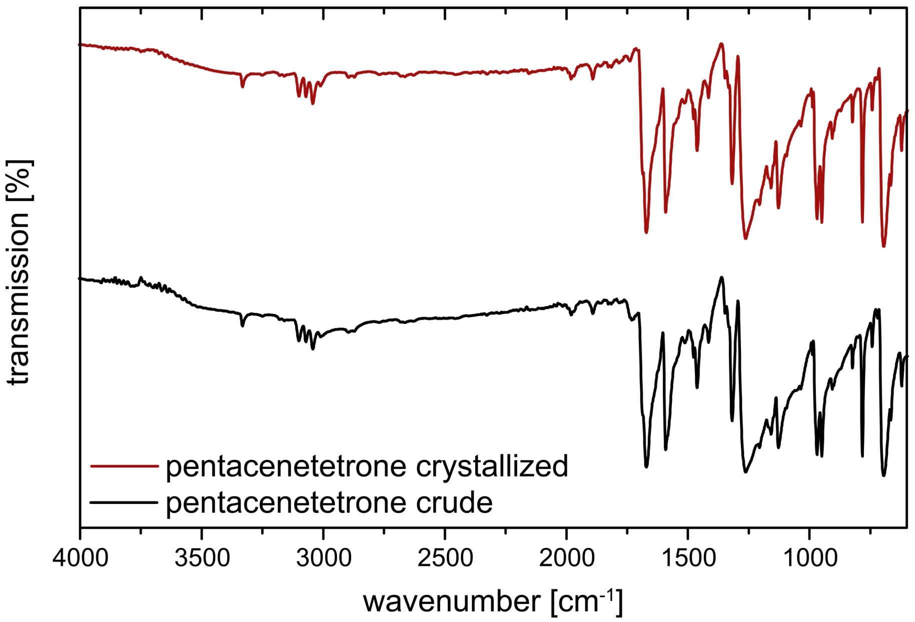

Figure S 32: ATR-FTIR spectra of pentacenetetrone before and after HT treatment (at T_R = 250 °C, t_H = 10 min and t_R = 15 min): both spectra are identical, which indicates that pentacenetetrone is stable under HT conditions.

The ^{1}H-NMR spectra before and after HT treatment (at T_R = 250 °C, t_H = 10 min and t_R = 15 min) of pentacenetetrone (Figure S 34) are completely identical, which confirms the above made assumption that it does not decompose (at least at the tested t_R of 15 min) under HT conditions. Neither new peaks occur nor the integral values change. Due to its extremely poor solubility in DMSO-d$_6$ the ^{1}H-NMR spectra of indigo (Figure S 33) before and after HT treatment (at T_R = 250 °C, t_H = 10 min and t_R = 15 min) show a bad signal/noise ratio. Nevertheless, one is able to find the corresponding literature signals of indigo in both spectra:[viii]

^{1}H-NMR: δ [ppm] (400 MHz, DMSO-d$_6$) = 7.60 (d, 2 H), 7.50 (t, 2 H), 7.32 (d, 2 H), 6.94 (t, 2 H).

However, for the crude (commercially purchased) product additional signals at 6.54 ppm and 7.07 ppm can be found (highlighted by blue boxes), which originate from an unknown impurity. For the crystallized product these signals are significantly less intense. Hence, in the case of indigo HT treatment even allows for removing these.

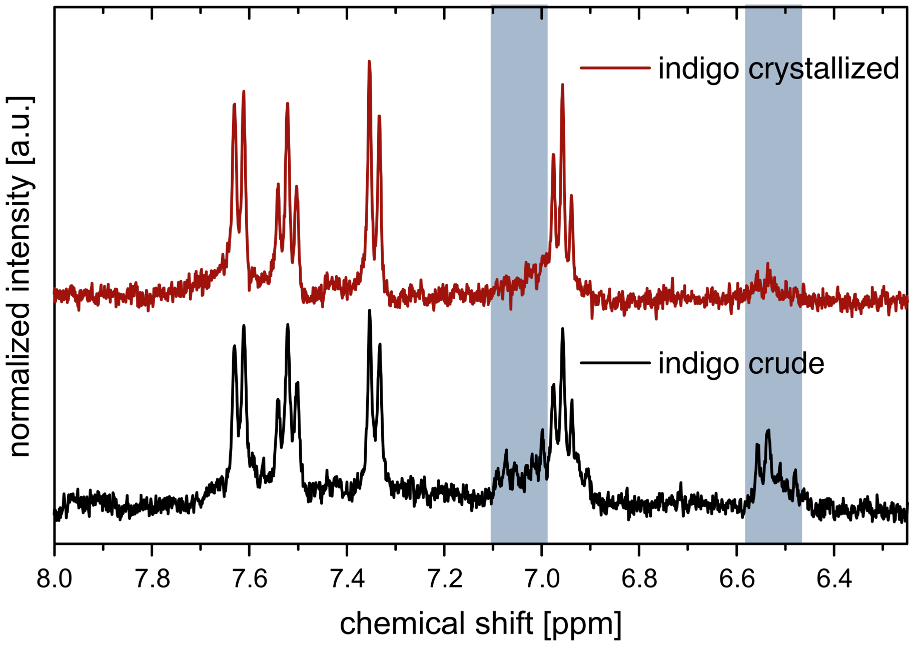

Figure S 33: ^{1}H-NMR spectra (measured in DMSO-d$_6$) of indigo before and after HT treatment (at T_R = 250 °C, t_H = 10 min and t_R = 15 min): crude indigo contains impurities (highlighted by blue boxes); the corresponding signals are significantly decreased after HT treatment.

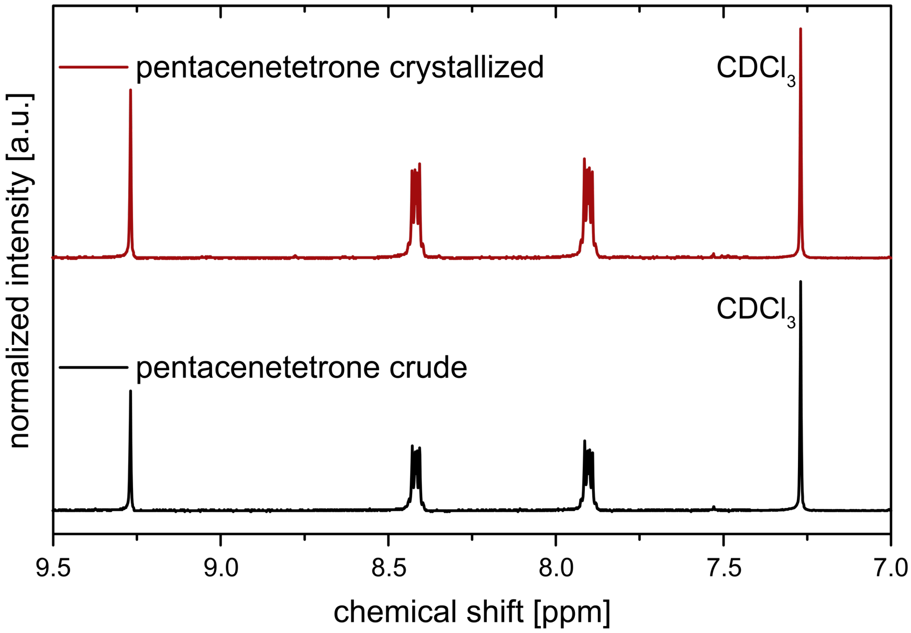

Figure S 34: ^{1}H-NMR spectra (measured in $CDCl_3$) of pentacenetetrone before and after HT treatment (at T_R = 250 °C, t_H = 10 min and t_R = 15 min): both spectra are identical, which indicates that pentacenetetrone is stable under HT conditions.

The PXRD patterns (Figure S 35 and Figure S 36) of the two tested substances (indigo and pentacenetetrone) after HT treatment (at T_R = 250 °C, t_H = 10 min and t_R = 15 min) show the same reflections as the ones before subjecting the compounds to HT conditions. However, this does definitely not mean, that indigo and pentacenetetrone do not dissolve and crystallize within the HT treatment process. Taking a closer look, one can see that HT treatment leads to sharper reflections, which is especially very well visible for pentacenetetrone. This is already a strong indication that indigo and

pentacenetetrone are not only stable under HT conditions, but that also their HT crystallization is indeed possible. Since the commercially purchases compounds are already highly crystalline – which is to be expected for low molecular weight compounds – only a minor increase in crystallinity can generally be achieved.

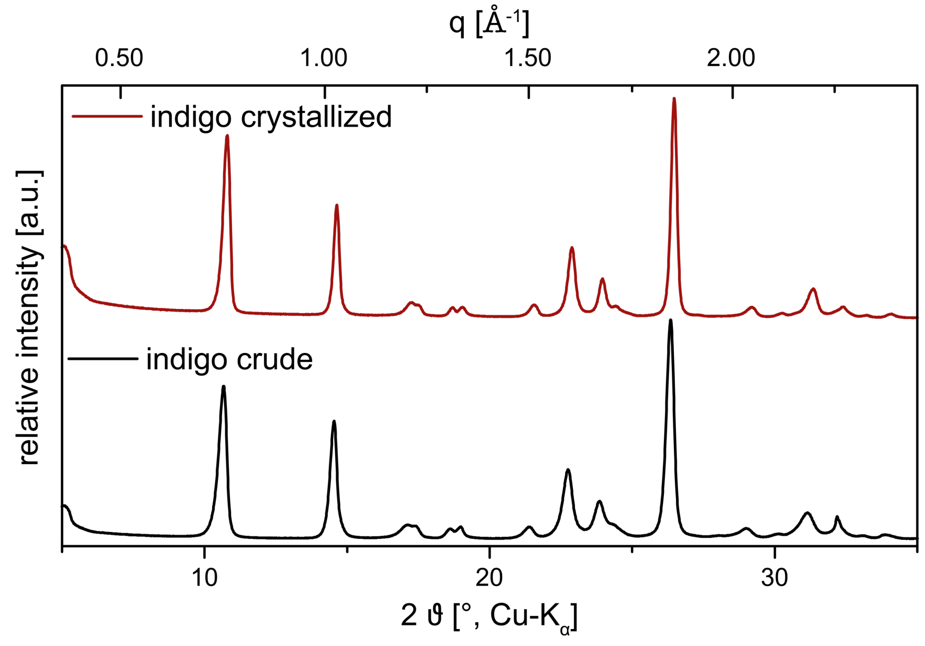

Figure S 35: PXRD patterns of indigo before and after HT treatment (at T_R = 250 °C, t_H = 10 min and t_R = 15 min): both diffractograms show the same reflections; HT treatment leads to slightly sharper and more pronounced reflections.

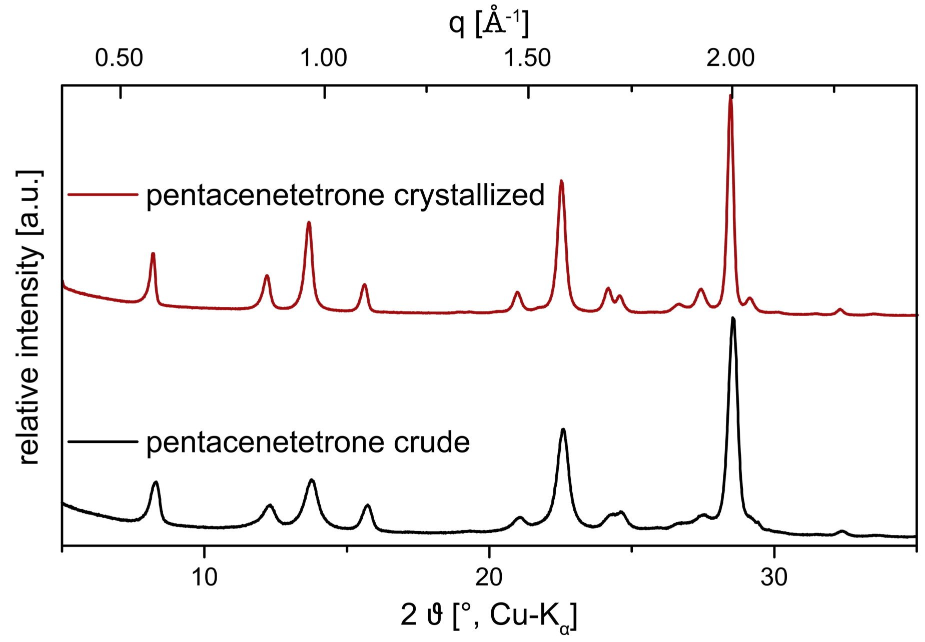

Figure S 36: PXRD patterns of pentacenetetrone before and after HT treatment (at T_R = 250 °C, t_H = 10 min and t_R = 15 min): both diffractograms show the same reflections; HT treatment leads to sharper and more pronounced reflections.

In order to completely clarify if it is possible to dissolve and crystallize indigo and pentacenetetrone *via* HT treatment morphologies before and after subjecting the three substances to HT conditions have to be compared.
Figure S 37 shows SEM micrographs of indigo before and after HT treatment (at T_R = 250 °C, t_H = 10 min and t_R = 15 min). Significant morphological differences can be found. The initially present roundish agglomerates of small particles (approximately 0.5 – 2 µm in length) are transformed into long, well-pronounced, angular needles (around 20 – 40 µm µm in length) that are sparsely decorated with smaller particles.

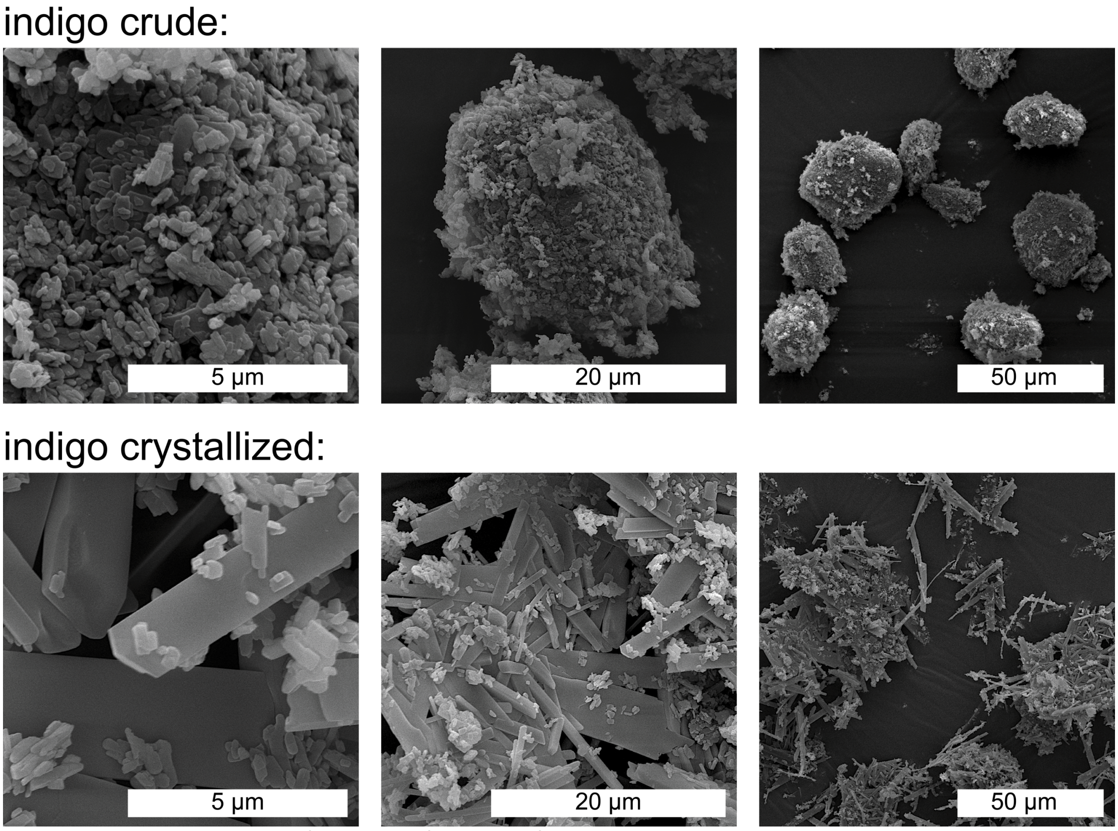

Figure S 37: SEM micrographs of indigo before and after HT treatment (at T_R = 250 °C, t_H = 10 min and t_R = 15 min): HT treatment transforms the initially present agglomerates of small particles (approximately 0.5 – 2 µm in length) into elongated needles (around 20 – 40 µm µm in length).

The SEM micrographs of pentacenetetrone before and after HT treatment (at T_R = 250 °C, t_H = 10 min and t_R = 15 min) are shown in Figure S 38. Similar to the previous case, the morphologies of crude pentacenetetrone and the crystallized product show non-negligible differences. While the crude compound is composed of angular shaped particles (30 – 100 µm in length) that are randomly decorated with smaller, needle-like particles, the crystallized product contains larger, angular particles (mainly 150 – 400 µm) that are decorated with small platelates. These platelates show some kind of preferential orientation and alignment.

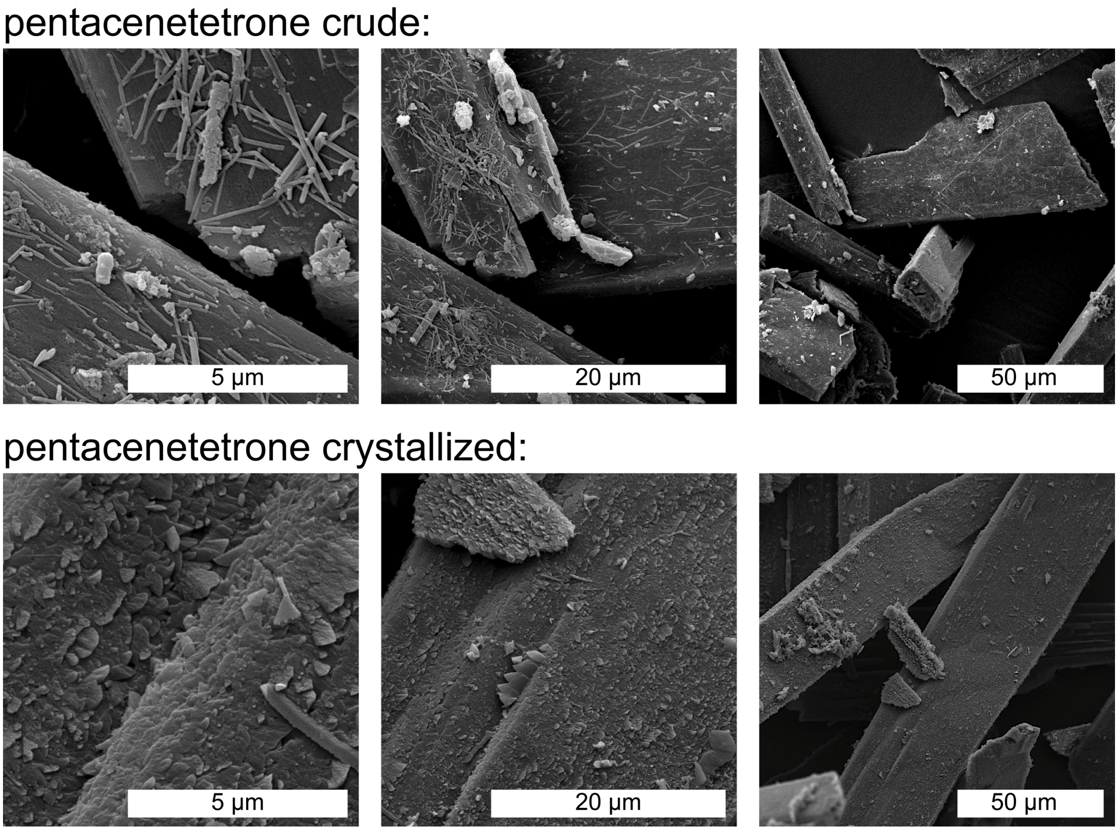

Figure S 38: SEM micrographs of pentacenetetrone before and after HT treatment (at T_R = 250 °C, t_H = 10 min and t_R = 15 min): HT treatment transforms the initial particles (approximately 30 -100 µm in length) with random surface texture into larger particles (mainly 150 – 400 µm µm in length) with an ordered surface texture.

6 References

[i] Manukian, B. K. (1969). Über Pyromellitsäure-und Cumidinsäure-Derivate. XIV. Teil [1]. Imidazole aus Pyromellitsäure-dianhydrid. *Helvetica Chimica Acta*, *52*(7), 2143-2150.

[ii] Mamada, M., Pérez-Bolívar, C., & Anzenbacher Jr, P. (2011). Green synthesis of polycyclic benzimidazole derivatives and organic semiconductors. *Organic letters*, *13*(18), 4882-4885.

[iii] Cordes, M. M., & Walter, J. L. (1968). Infrared and Raman studies of heterocyclic compounds—II Infrared spectra and normal vibrations of benzimidazole and bis-(benzimidazolato)-metal complexes. *Spectrochimica Acta Part A: Molecular Spectroscopy*, *24*(9), 1421-1435.

[iv] Loutfy, R. O., Hor, A. M., Kazmaier, P. M., Burt, R. A., & Hamer, G. K. (1991). Organic photoconductive (OPC) devices incorporating bisarylimidazole perinone pigments. *Dyes and Pigments*, *15*(2), 139-156.

[v] Teteruk, J. L., Glinnemann, J., Heyse, W., Johansson, K. E., van de Streek, J., & Schmidt, M. U. (2016). Local structure in the disordered solid solution of cis-and trans-perinones. *Acta Crystallographica Section B: Structural Science, Crystal Engineering and Materials*, *72*(3), 416-433.

[vi] Mizuguchi, J. (2004). Crystal structure and electronic characterization of trans-and cis-perinone pigments. *The Journal of Physical Chemistry B*, *108*(26), 8926-8930.

[vii] Laschat, S., Baro, A., Steinke, N., Giesselmann, F., Hägele, C., Scalia, G., & Tosoni, M. (2007). Discotic liquid crystals: from tailor-made synthesis to plastic electronics. *Angewandte Chemie International Edition*, *46*(26), 4832-4887.

[viii] Guengerich, F. P., Sorrells, J. L., Schmitt, S., Krauser, J. A., Aryal, P., & Meijer, L. (2004). Generation of new protein kinase inhibitors utilizing cytochrome P450 mutant enzymes for indigoid synthesis. Journal of medicinal chemistry, 47(12), 3236-3241.
